# Identification of multiple genomic DNA sequences which form i-motif structures at neutral pH

**DOI:** 10.1093/nar/gkx090

**Published:** 2017-02-09

**Authors:** Elisé P. Wright, Julian L. Huppert, Zoë A. E. Waller

**Affiliations:** 1 School of Pharmacy, University of East Anglia, Norwich Research Park, Norwich NR4 7TJ, UK; 2 Intellectual Forum, Jesus College, University of Cambridge, Cambridge CB5 8BL, UK; 3 Centre for Molecular and Structural Biochemistry, University of East Anglia, Norwich Research Park, Norwich NR4 7TJ, UK

## Abstract

i-Motifs are alternative DNA secondary structures formed in cytosine-rich sequences. Particular examples of these structures, traditionally assumed to be stable only at acidic pH, have been found to form under near-physiological conditions. To determine the potential impact of these structures on physiological processes, investigation of sequences with the capacity to fold under physiological conditions is required. Here we describe a systematic study of cytosine-rich DNA sequences, with varying numbers of consecutive cytosines, to gain insights into i-motif DNA sequence and structure stability. i-Motif formation was assessed using ultraviolet spectroscopy, circular dichroism and native gel electrophoresis. We found that increasing cytosine tract lengths resulted in increased thermal stability; sequences with at least five cytosines per tract folded into i-motif at room temperature and neutral pH. Using these results, we postulated a folding rule for i-motif formation, analogous to (but different from) that for G-quadruplexes. This indicated that thousands of cytosine-rich sequences in the human genome may fold into i-motif structures under physiological conditions. Many of these were found in locations where structure formation is likely to influence gene expression. Characterization of a selection of these identified i-motif forming sequences uncovered 17 genomic i-motif forming sequence examples which were stable at neutral pH.

## INTRODUCTION

DNA can fold into a range of non-duplex secondary structures that can contribute to the function and transcription of genes. For example, guanine-rich DNA sequences can form G-quadruplexes, structures that are well characterized and have documented transcriptional consequences (1–3). G-quadruplex forming sequences are found in telomeric regions and throughout the genome, particularly oncogene promoters (4), where it is thought that folded structures can affect protein interactions with DNA (5). These G-quadruplex structures have been shown to form in human cells, using a fluorescent antibody specific to the folded structures (6).

The complementary strand to a G-quadruplex forming region is obviously cytosine-rich. It has been known for some time that some such sequences are also able to fold into another secondary structure called an i-motif (7). This conformation relies on hemi-protonated cytosine:cytosine base pairs to hold two intercalated hairpins in a quadruplex structure (7). The stability of i-motifs depends on the length of the cytosine tract, loop sequences, temperature, salt concentration, sequence length and environmental pH (8–11). While i-motifs are far more stable at acidic pH, due to the requirement for hemi-protonation of the cytosines, a few i-motifs can form at neutral pH with a free H^+^ ion being taken up upon formation of the folded structure (12). These have been demonstrated at low temperatures (4°C) (13), under molecular crowding conditions, (14) under conditions of negative superhelicity (15) and in the presence of silver(I) cations (16). There is also an example of an i-motif forming sequence from the promoter region of HIF-1α which shows unexpected stability near neutral pH; the transitional pH (*pH*_T_), where the sequence is 50% folded, was found to be 7.2 (17). Given that G-quadruplexes are found in gene promoters, especially oncogenes, as well as telomeric regions (4), it is reasonable to suggest that i-motif formation could also play a role in regulation, either as a lead element or by stabilizing the G-quadruplex form relative to duplex. This would make i-motifs unique and viable targets for chemical intervention in biology, especially where i-motifs can directly influence transcription.

The structures of i-motifs are arguably more complex than G-quadruplexes, and it has been suggested that an equilibrium exists between i-motif, hairpin and duplex. This has been probed in a system where i-motif structures have been implicated in modulation of transcription (18–20). Here it was shown that small molecule ligands IMC-48 and IMC-76 can influence the regulation of BCL-2 gene expression by interacting with the i-motif forming sequences in BCL-2 (18–20). Further evidence for the occurrence of a functional i-motif in the MYC promoter was recently disclosed, highlighting the importance of negative superhelicity, the binding of proteins hnRNP K and hnRNP LL and unfolding of the i-motif structure for transcriptional activation (21). Furthermore, stabilization of the i-motif forming sequence in the human telomere by carboxyl-modified single walled carbon nanotubes has also been shown to inhibit telomerase activity and interfere with the telomere functions in cancer cells (22). This suggests that the i-motif is not just a structural curiosity, or only useful for biotechnological applications, but is able to be targeted by ligands or proteins to alter biological responses. Nevertheless, evidence for the existence of i-motifs has not yet been directly observed in cells or *in vivo*. A more detailed investigation to aid definition of potentially stable i-motifs is required to eventuate this kind of study (8,11). Better characterization of i-motif structures has a bearing on their already well-established use as biocompatible pH sensors, vehicles for drug delivery, nanotube diagnostics, logic gate components and nanocontainers that respond to their environment (23–26). These nanomachines are driven by the thermal stability and pKa of the sequences of DNA which can form i-motifs (11).

In order to facilitate studies of i-motifs, both in the human genome and for biotechnological applications, more information about what variables influence their formation is required. Previous work examining uncomplicated cytosine-rich model sequences focused on the altered thermodynamic parameters and it was found that increased *T*_m_ was caused by lengthened cytosine tracts (12). Herein we build from this study, investigating the stability and folding potential of different i-motifs by extending the range of cytosine tract lengths in model sequences and also i-motif forming sequences from the human genome. A number of biophysical methods were used to characterize the oligonucleotides (ODNs) including ultraviolet (UV) melting experiments, thermal difference spectroscopy, circular dichroism (CD) and native polyacrylamide gel electrophoresis. This approach will allow us to distinguish which sequences are likely to fold under physiological conditions, and hence more likely to have a biological function.

## MATERIALS AND METHODS

### Oligonucleotides

ODNs were supplied by Eurogentec (Belgium), synthesized on a 40, 200 or 1000 nmol scale and purified by either reverse phase HPLC, or in the longer sequences (>50 bases), polyacrylamide gel electrophoresis (PAGE). Samples were dissolved in MilliQ water to give 100 μM final concentrations and confirmed using a Nanodrop. Each ODN in the library consisted of four cytosine tracts separated by three loops containing thymines. The notation C_n_T_x_ is used where *n* = the number of cytosines in each of four tracts and *x* = the number of thymines in each of the three loops. A summary of the model ODNs used in this study is given in Table 1. A sample of 33 genomic i-motif forming sequences from the Quadparser screen were selected to represent the variety of sequences identified including different lengths (23–116 bases), chromosomal positions and from both coding and non-coding strands (Table 2 and Supplementary Table S3). For all experiments, ODNs were diluted in buffer containing 10 mM sodium cacodylate and 100 mM sodium chloride at the pH as detailed, thermally annealed by heating in a heat block at 95°C for 5 min and cooled slowly to room temperature overnight.

**Table 1.** Library of oligonucleotides sequences and data for their melting temperature (***T*_m_**), annealing temperature (***T*_a_**) and transitional pH (**pH_T_**)

Sequence 5΄ - 3΄	Bases	Notation	pH 5.5		pH 7.4		*pH*_T_
			*T*_m1_/*T*_m2_	*T*_a_	*T*_m1_/*T*_m2_	*T*_a_	
TAA(C_3_)_4_	24	hTeloC	43 ± 0.0	41 ± 0.6	nd	nd	6.5
C(T_3_C)_3_	13	C_1_T_3_	nd	nd	nd	nd	nd
C_2_(T_3_C_2_)_3_	17	C_2_T_3_	27.1 ± 1.0	26.2 ± 0.6	nd	nd	6.1
C_3_(T_3_C_3_)_3_	21	C_3_T_3_	46.3 ± 1.8	45.5 ± 0.6	7 ± 0.6	6 ± 0.0	6.7
C_4_(T_3_C_4_)_3_	25	C_4_T_3_	56.4 ± 1.2	55.6 ± 0.6	15.8 ± 0.7	8.4 ± 0.2	7.1
C_5_(T_3_C_7_)_5_	29	C_5_T_3_	61.4 ± 0.6	60.6 ± 0.0	26.2 ± 1.8	6.7 ± 0.6	7.2
C_6_(T_3_C_6_)_3_	33	C_6_T_3_	65.5 ± 0.6	63.6 ± 0.0	9.1 ± 3.1/31.3 ± 0.6	9.7 ± 0.5	6.8
C_7_(T_3_C_7_)_3_	37	C_7_T_3_	68.5 ± 0.6	64.7 ± 0.0	12.8 ± 1.2/32 ± 1.2	8.7 ± 1.0	7.4
C_8_(T_3_C_8_)_3_	41	C_8_T_3_	72.6 ± 0.0	64.7 ± 0.0	18.8 ± 0.8/35 ± 2.0	13.8 ± 0.7	7.1
C_9_(T_3_C_9_)_3_	45	C_9_T_3_	75.6 ± 1.8	65.7 ± 0.6	20.8 ± 1.8/35 ± 1.7	12.1 ± 0.6	7.3
C_10_(T_3_C_10_)_3_	49	C_10_T_3_	66 ± 0.7/77 ± 2.7	65 ± 0.4	21.9 ± 1.9/41.1 ± 0.2	18.2 ± 0.0	7.3
C_5_(T_1_C_5_)_3_	23	C_5_T_1_	60.6 ± 1.5	59.6 ± 0.0	15.8 ± 0.2	14.1 ± 0.6	6.9
C_5_(T_2_C_5_)_3_	26	C_5_T_2_	60.6 ± 0.6	60.6 ± 0.0	25.2 ± 0.6	16.1 ± 0.0	7.1
C_5_(T_4_C_5_)_3_	32	C_5_T_4_	63.3 ± 0.2	60.6 ± 0.6	29.3 ± 0.0	5.3 ± 0.6	6.7

**Table 2.** Thermal and pH stability of the genomic i-motif candidate oligonucleotides used in this study

			pH 7.0		
Notation	Bases	Sequence 5′ - 3′	*T*_m1_/*T*_m2_	*T*_a_	*pH_T_*
AC017019.1	28	CCC-CCC-TCC-CCC-CCT-CCC-CCC-TCC-CCC-C	27.9 ± 0.6	18.0 ± 0.2	7.1
AC018878.3	26	CCC-CCA-CCC-CCA-GCC-CCC-TTT-CCC-CC	18.1 ± 0.2	7.5 ± 0.5	7.1
ATXN2L	24	CCC-CCC-CCC-CCC-CCC-CCC-CCC-CCC	23.7 ± 1.0	22.5 ± 0.6	7.0
CAMK2G	50	CCC-CCA-GGC-CCC-GCC-AGT-CCC-CCC-CCC-CGC-CCG-GCC-CCC-GGC-CCG-CCC-CC	13.0 ± 1.2	11.3 ± 0.7	6.9
DAP	29	CCC-CCG-CCC-CCG-CCC-CCG-CCC-CCG-CCC-CC	24.7 ± 0.5	22.0 ± 0.4	7.0
DRP2	70	CCC-CCT-CTT-CCC-CTC-TCC-CCC-TCT-CCC-CCT-CTC-TCC-CTC-TTC-CCC-CTC-TCC-TTG-TCT-CCTTCT-CTC-CCC-C	4.5 ± 0.7	6.5 ± 0.7	6.0
DUX4L22	41	CCC-CCG-AAA-CGC-GCC-CCC-CTC-CCC-CCT-CCC-CCC-TCT-CCC-CC	29.2 ± 0.2	14.2 ± 0.2	7.1
GH2	42	CCC-CCA-CCC-CCA-CCC-CCA-TCC-CCA-CGC-CCC-GCC-CCC-GCC-CCC	22.7 ± 1.0	15.2 ± 0.9	7.1
HIC2	74	CCC-CCG-GGA-CAG-GGA-CCC-TGG-CCC-CCC-CCG-ACA-GGC-TGA-CGC-CCA-CCC-CCT-CAA-ACT-CTG-GTG-GAC-TTA-CCC-CC	7.5 ± 2.1	7.8 ± 2.0	6.4
HOXC10	24	CCC-CCA-CCC-CCA-CCC-CCA-CCC-CCC	17.7 ± 0.8	14.0 ± 0.2	7.1
HOXD10	26	CCC-CCC-CCC-CCT-CCC-CCG-CGG-CCC-CC	10.2 ± 0.9	5.2 ± 0.2	7.1
JAZF1	31	CCC-CCC-CCG-CCC-CCG-CCC-CCG-CCC-TCC-CCC-C	20.4 ± 0.4	18.5 ± 0.6	7.1
MSMO1	23	CCC-CCG-CCC-CCG-CCC-CCG-CCC-CC	16.6 ± 0.5	15.9 ± 0.4	6.7
NFATC1	45	CCC-CCG-TTT-CCC-CCG-CCA-GCC-CCA-GCG-CCC-CCC-TGC-CCG-GCC-CCC	23.2 ± 0.0/28.8 ± 0.5	18.8 ± 0.6	7.1
PIM1	45	CCC-CCG-ACG-CGC-CCC-CCA-ACA-CAC-AAA-CCC-CCA-GAA-TCC-GCC-CCC	29.4 ± 1.9	5.1 ± 0.2	7.0
PLCB2	36	CCC-CCG-CCT-CTT-CTG-GAG-GCC-CCC-GCC-CCC-ACC-CCC	15.0 ± 0.2	13.2 ± 0.2	7.0
QSOX1	25	CCC-CCG-CCC-CCG-AGC-CCC-CGC-CCC-C	20.1 ± 0.2	11.9 ± 0.4	7.1
RAE1	116	CCC-CCC-GCC-CCC-CCC-GCC-CCC-CCG-CGC-CGC-CCC-CCC-CCG-CCC-CCC-GCC-CCC-GTC-CCC-CCG-CCC-CCC-CCG-CCC-CCC-CCG-CCC-CCC-GTC-CCC-CCG-CCC-CCC-CGC-CCC-CCC-GTC-CCC-CC	27.1 ± 7.4	13.6 ± 6.3	6.8
RUNX1–1	31	CCC-CCC-CCG-CAC-CCC-TTC-CCC-CGG-CCC-CCC-C	14.3 ± 0.9/25.0 ± 0.4	9.8 ± 0.4	6.7
RUNX1–2	36	CCC-CCC-TCC-CCC-TGC-CTC-TCC-CTC-CCC-CCT-TTC-CCC	13.2 ± 0.2/24.4 ± 0.2	10.1 ± 0.0	6.5
RUNX1–3	32	CCC-CCC-TTT-CCC-CTG-CCC-CCC-CTG-CCT-CCC-CC	10.7 ± 0.6/26.2 ± 0.0	9.7 ± 0.6	6.7
SHANK1b	28	CCC-CCC-TCC-CCC-CAC-CCC-CCA-CCC-CCC-C	22.5 ± 0.6	12.0 ± 0.0	7.1
SHANK3	80	CCC-CCG-CCT-CCG-GCG-CAG-CCC-CCT-CGC-CAC-CCC-CGC-TTC-CCT-CCC-GTC-TCA-GGC-CCC-CTC-CCC-CCG-CCG-CCC-CCG-CCC-CC	18.3 ± 1.9	5.6 ± 1.0	6.6
SHANK3b	79	CCC-CCC-GCA-CCG-AGG-CCT-AGG-ACT-CCC-CCC-CCC-AAC-CCC-GTC-ACA-GCC-CCC-CAG-ACC-CCC-GCC-CCG-TGG-CTC-GGC-CCC-C	12.6 ± 3.6	4.8 ± 0.7	6.5
SNORD112	36	CCC-CCC-CCC-GCC-CCC-CAC-CCC-CCC-ACC-CCC-CCC-CCC	25.8 ± 0.5	15.9 ± 0.4	7.2
SOX1	57	CCC-CCT-GCA-GGC-CCC-CCT-GCG-CCT-CCC-CCC-CCC-CGC-CAC-TGG-CGC-CTG-GCT-TCC-CCC	9.0 ± 0.2	6.5 ± 0.5	6.9
STX17	33	CCC-CCG-CCC-CCG-CCC-CCG-CCC-CGC-AGG-GCC-CCC	19.5 ± 0.6	15.0 ± 0.2	7.0
Tandem Repeat (LA16c-OS12.2)	57	CCC-CCC-GTG-TCG-CTG-TTC-CCC-CCG-TGT-CGC-TGT-TCC-CCC-CGT-GTC-GCT-GTT-CCC-CCC	9.4 ± 1.5/31.6 ± 0.6	6.7 ± 0.6	6.6
TRABD	23	CCC-CCG-CCC-CCC-CCC-CCC-CCC-CC	21.3 ± 0.2	19.3 ± 0.2	6.9
WNT7A	48	CCC-CCG-CCC-CTC-CCT-CCT-TTC-CCC-CGT-CCC-TCC-CCC-GCC-CCC-TCC-CCC	22.7 ± 2.5	16.1 ± 0.0	7.1
ZBTB7B	58	CCC-CCC-ATC-CCT-CCC-CTC-CCT-CCC-CCC-GCC-CCT-GCC-ACC-CCC-CAA-ACT-CCC-CCC-CCC-C	25.5 ± 1.3	10.0 ± 0.2	7.1
ZFP41	52	CCC-CCA-GCC-CCC-GCC-GAC-CCC-CAG-CTC-CCG-CCT-CCG-CCG-ACC-CCC-AGC-CCC-C	21.7 ± 0.7/35.6 ± 0.4	17.4 ± 0.4	7.0
ZNF480	23	CCC-CCG-CCC-CCG-CCC-CCG-CCC-CC	17.1 ± 0.0	15.2 ± 0.2	6.7

### UV absorption spectroscopy

UV spectroscopy experiments were performed on a Cary 60 UV-Vis spectrometer (Agilent Technologies) equipped with a TC1 Temperature Controller (Quantum Northwest) and recorded using a low volume masked quartz cuvette (1 cm path length). ODNs (Tables 1 and 2) were diluted to 2.5 μM in buffer at the desired pH. Samples (200 μl) were transferred to a cuvette, covered with a layer of silicone oil and stoppered to reduce evaporation of the sample. The absorbance of the ODN was measured at 295 nm as the temperature of the sample was held for 10 min at 4°C then heated to 95°C at a rate of 0.5°C per min, held at 95°C before the process was reversed; each melting/annealing process was repeated three times. Data were recorded every 1°C during both melting and annealing and each point was the average of three scans. Melting temperatures (*T*_m_) were determined using the first derivative method. Thermal difference spectra (TDS) were calculated by subtracting the spectrum between 220 and 320 nm of the folded structure at 4°C from that of the unfolded structure at 95°C. The data was normalized and the maximum change in absorption was set to +1 as previously described (27).

### Circular dichroism

CD spectra were recorded on a Jasco J-810 spectropolarimeter using a 1 mm path length quartz cuvette. ODNs (Tables 1 and 2) were diluted to 10 μM (total volume: 200 μl) in buffer at pH increments of 0.5 pH unit from 4.0 to 8.0. The scans were recorded at room temperature (20°C) between 200 and 320 nm. Data pitch was set to 0.5 nm and measurements were taken at a scanning speed of 200 nm/min, response time of 1 s, bandwidth of 2 nm and the 100 mdeg sensitivity; each spectrum was the average of three scans. Samples containing only buffer were also scanned according to these parameters to allow for blank subtraction. Transitional pH for i-motif was calculated from the inflection point of fitted ellipticity at 288 nm. Final analysis and manipulation of the data was carried out using Origin 2015.

### Native polyacrylamide gel electrophoresis

Acrylamide gels (20%) were buffered with 50 mM tris, 50 mM HEPES at pH 7.4. ODNs were annealed in buffer at pH 7.4. Concentrated loading buffer (glycerol 50% (w/v), 0.5 M tris, 0.5 M HEPES pH 7.4) was then added and the samples loaded onto the gel for electrophoresis at 50 V for 5 h at room temperature. Once complete, gels were stained overnight without shaking in 50 mg/ml Stainsall (Sigma) dissolved in 10% (v/v) formamide, 25% (v/v) isopropanol and 15 mM tris. Gels were rinsed in water twice before being destained via light exposure. Gels were then imaged using a Gel Doc XR+ System (BioRad).

### Genome searching

The reference human genome (NCBI Build 38) was downloaded from Ensembl (28). Quadparser was used to identify potential i-motif sequences on either strand of the sequence (29) using the parameters: GC 5 4 1 19. Transcription start site locations and Gene Ontology codes were also downloaded from Ensembl using the Ensmart tool. Potential i-motif sequences were screened for their proximity to transcriptional start sites and genes matched to GO codes using custom perl code.

## RESULTS AND DISCUSSION

To enable an indication of which sequences were more likely to be stable at neutral pH, a methodical examination of the sequence parameters for i-motif formation was performed. We used biophysical techniques to characterize a model cytosine-rich sequence library with variable tract lengths. UV spectroscopy was used to determine both the melting (*T*_m_) and annealing temperatures (*T*_a_) for each of the ODNs. All of the sequences in our model library were thermally annealed and melted in 10 mM sodium cacodylate with 100 mM sodium chloride at both pH 5.5 and 7.4. These pH conditions were selected to offer an optimal acidic environment for i-motif formation (5.5) and a physiological pH for comparison (7.4).

At pH 5.5, all of these ODNs, except for C_1_T_3_ and C_10_T_3_, showed two state (folded and unfolded) melting and annealing curves (Supplementary Figure S1). The UV melting experiment indicated that C_1_T_3_ did not form a folded structure, even at low temperature (Supplementary Figure S1A). In contrast, C_10_T_3_, showed a two-stage UV melt, indicating the presence of structures with two different melting points (Supplementary Figure S1J). Given the length of these sequences, this may be due to kinetic factors, where it will take longer to complete formation of one single structure; a combination of unimolecular and multimeric species or evidence of a folded hairpin structure in addition to i-motif may have arisen (30).

**Figure 1.**
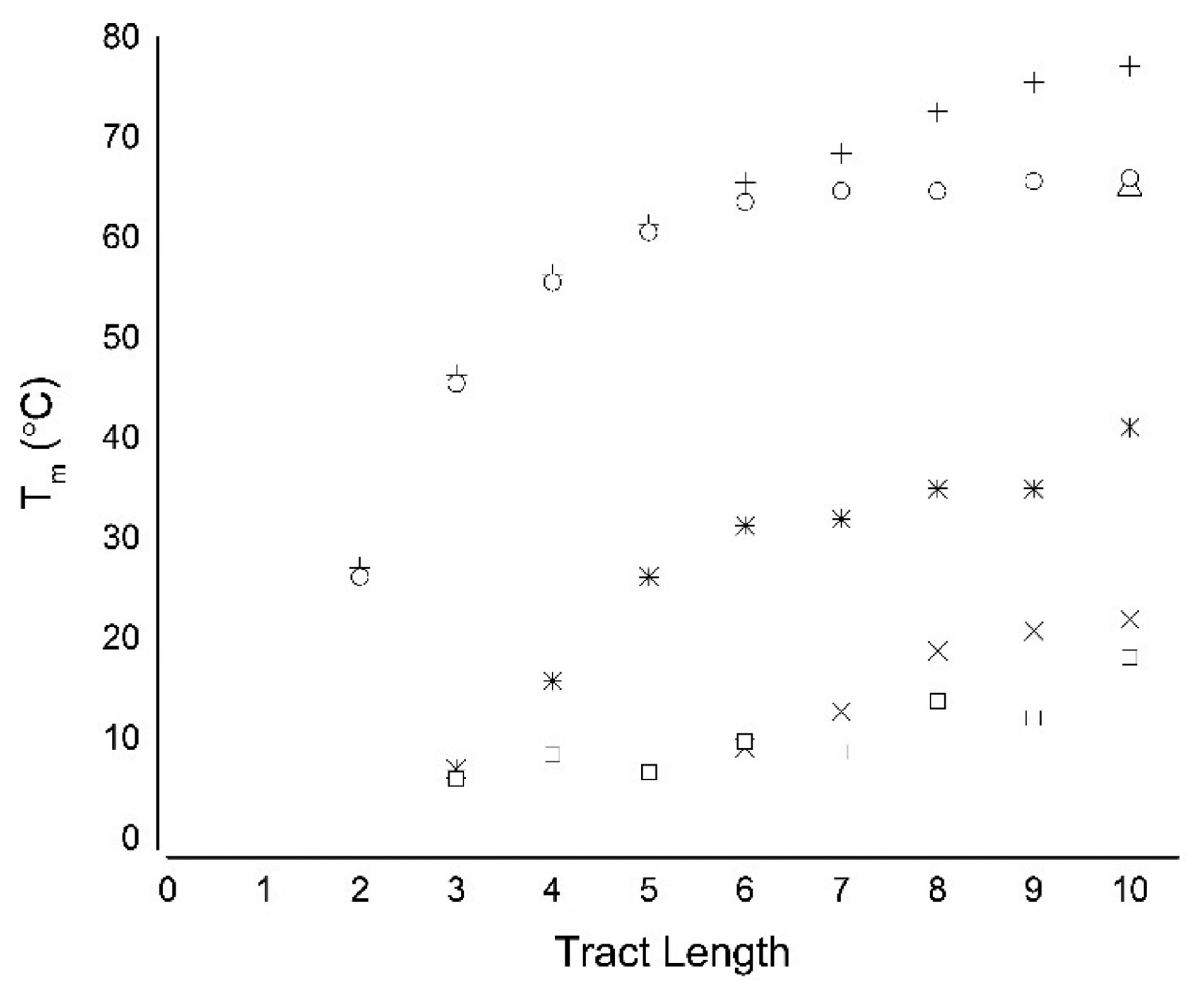
ODN stability with increasing cytosine tract length. ODNs (2.5 μM) were annealed in 10 mM sodium cacodylate with 100 mM sodium chloride at the indicated pH. + pH 5.5 *T*_m1_; Δ pH 5.5 *T*_m2_ (10 cytosine tract only); o pH 5.5 *T*_a_; 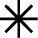
pH 7.4 *T*_m1_; × pH 7.4 *T*_m2_; and ■ pH 7.4 *T*_a_.

At pH 7.4, a minimum tract length of three cytosines was required to observe folded and unfolded states under the experimental conditions; C_3_T_3_, C_4_T_3_ and C_5_T_3_ all show a single UV melting transition between these two states (Supplementary Figure S2C–E). However, at pH 7.4, ODNs with cytosine tracts greater than or equal to six nucleobases showed two-stage melting curves (Supplementary Figure S2F–J). This suggests the presence of two distinct folded species at pH 7.4. Under both pH conditions the *T*_m_ was found to increase with increasing number of cytosines per tract (Figure 1). In ODNs with shorter cytosine tract lengths, the increase in temperature with each additional cytosine was pronounced, but after tract lengths of five, this was reduced. For example at pH 7.4, the increase in *T*_m_ from 3 to 4 cytosines per tract was 9°C, 4 to 5 was 10°C whereas the increases thereafter are smaller: 5 to 6 was 5°C, 6 to 7 was 1°C and 8 to 9 was 3°C. The annealing temperatures of these ODNs showed a similar pattern; as additional cytosines were added to the tracts, the annealing temperature also increased (Table 1 and Figure 1). However for ODNs with 6 or more cytosines per tract this increase was marginal and the change in annealing temperature plateaued.

**Figure 2.**
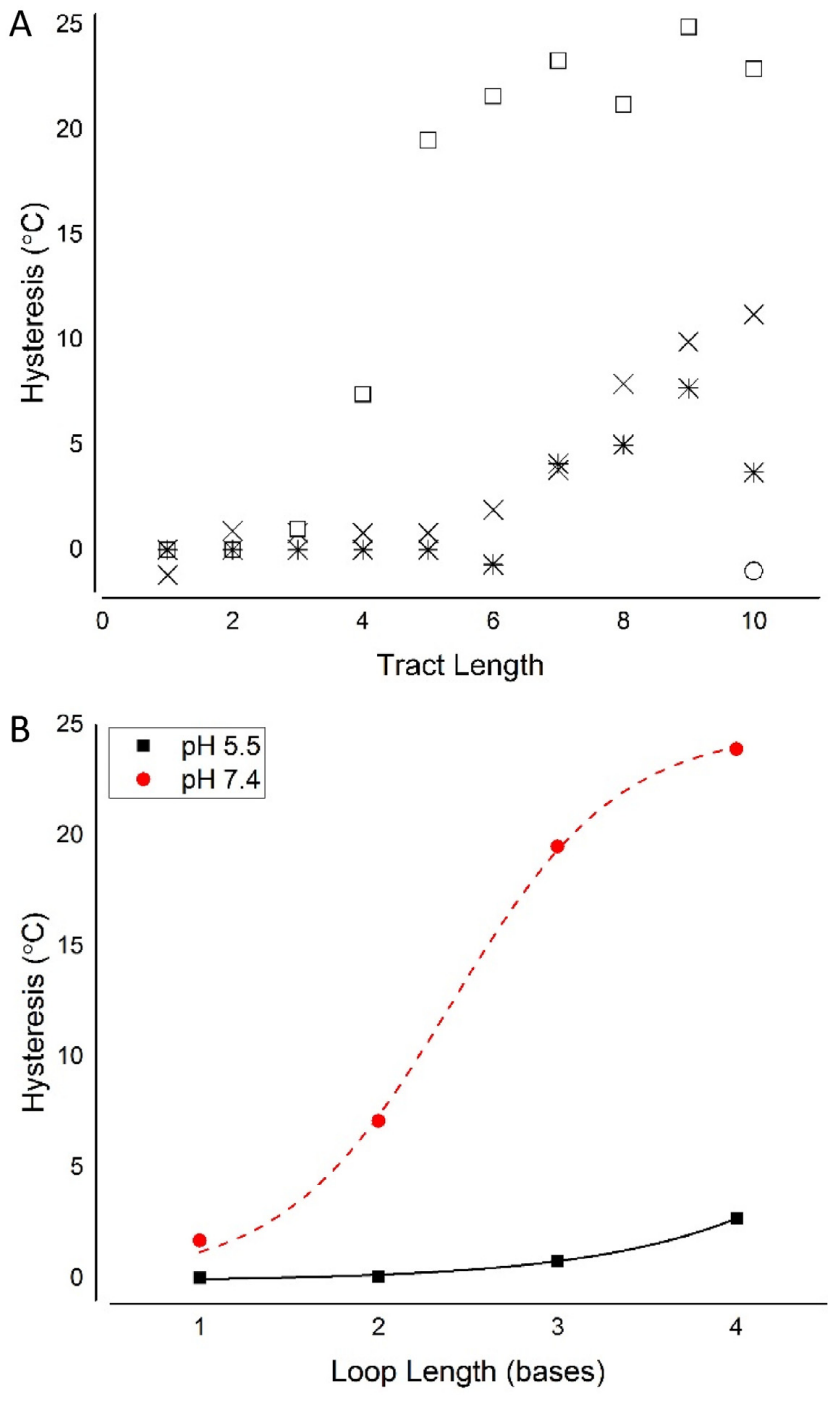
The effects of cytosine tract length (**A**) and loop length (**B**) on hysteresis. ODNs (2.5 μM) were annealed in 10 mM sodium cacodylate with 100 mM sodium chloride at the indicated pH. (A) × Hysteresis at pH 5.5; o Secondary hysteresis at pH 5.5 (10 cytosine tract only); ■ Hysteresis at pH 7.4; 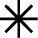
Secondary hysteresis at pH 7.4. (B) Hysteresis at pH 5.5 (▪) and at pH 7.4 (•) in ODNs containing tracts of 5 cytosines with increasing lengths of thymine loops.

The relationship between hysteresis and tract length was also examined. When a UV melting curve is not perfectly superimposable on its corresponding annealing curve, the difference between the curves reveals a hysteresis. The presence of a hysteresis suggested that association kinetics are slower than dissociation and that the folding process was complex (12). At both pH 5.5 and 7.4, as cytosine tract length increased beyond six cytosines, the hysteresis also increased. It is clear that the degree of hysteresis approached an upper limit in ODNs with cytosine tract lengths of six or more at pH 7.4 (Figure 2A). At pH 5.5, however, the size of the hysteresis was reduced compared to the analogous experiment at pH 7.4, indicative of the difference in kinetics in the presence of acid. At pH 7.4, formation of the folded structure was complex, the association kinetics were slower and more time was required to assemble the final structure. This suggested an upper limit in C-tract length for likely i-motif candidates in the genome. Slow kinetics of formation may hamper any potential regulatory function of genomic i-motif sequences, but may still be influenced by chemical intervention or the binding of proteins.

**Figure 3.**
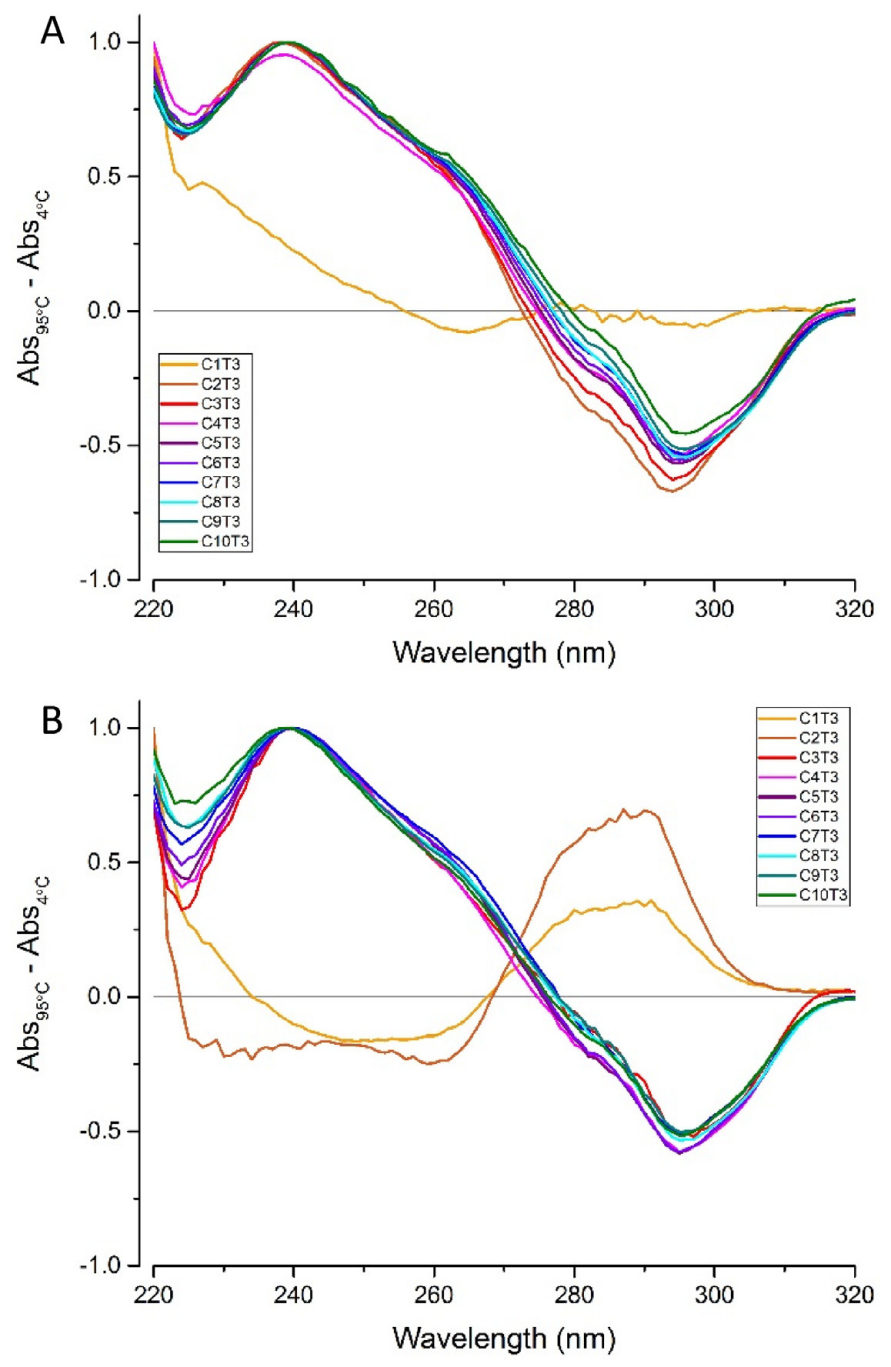
The thermal difference spectra calculated between 95 and 4°C of each of the oligonucleotides (ODNs) at pH 5.5 (**A**) and 7.4 (**B**). ODNs (2.5 μM) were annealed in 10 mM sodium cacodylate with 100 mM sodium chloride at the indicated pH.

Having established the presence of secondary structures by UV spectroscopy, TDS were determined to indicate the dominant folded species of each ODN at pH 5.5 and 7.4. DNA secondary structures absorb UV light differently when folded and unfolded and taking the difference between these spectra gives an indicative spectrum which can be used for characterization. The resultant difference spectra is characteristic of the folded DNA structure present at the lower temperature (27). At pH 5.5, with the exception of C_1_T_3_, the TDS of all the ODNs showed a positive peak at 240 nm and a negative peak at 295 nm. This was consistent with the TDS of the previously characterized folded human telomeric i-motif (hTeloC, Table 1) (27). This indicated that the ODNs with a tract length of 2 to 10 cytosines were folded into i-motif structures (Figure 3A) at pH 5.5. At pH 7.4, however, the ODNs with two cytosines or fewer per tract gave rise to a TDS that was consistent with random coil (Figure 3B). The TDS data indicated that the single stage melting curve of C_3-5_T_3_ at pH 7.4 (Supplementary Figure S2C–E) showed the unfolding of predominantly i-motif at 4°C. Despite the indication of a second species in the UV melts at pH 7.4, the TDS of ODNs C_6-10_T_3_ also showed features characteristic of i-motif: positive at 240 nm and negative at 295 nm (Figure 3). While the TDS data was not very useful for observing species in a mixed population, the dominant species was consistent with i-motif structure.

Additional UV-melts were carried out to ensure that the multistage UV melting curves in ODNs C_6-10_T_3_ at pH 7.4 could not be attributed to intermolecular interactions. The C_7_T_3_ ODN, as an example, has two melting temperatures (*T*_m_ = 12.8 and 33.0°C) and a single annealing (*T*_a_ = 8.7°C) (Supplementary Figure S2G). To confirm whether this effect was due to intermolecular interactions, a lower (1.25 μM) and higher (5 μM) concentration were also assessed using UV spectroscopy. The two-stage melting curves were visible at each of the concentrations and the shape of each of the curves were comparable (Supplementary Figure S3). Overlaying the normalized melting curves did not show a concentration dependent change in melting temperature, indicating predominantly intramolecular folded species.

**Figure 4.**
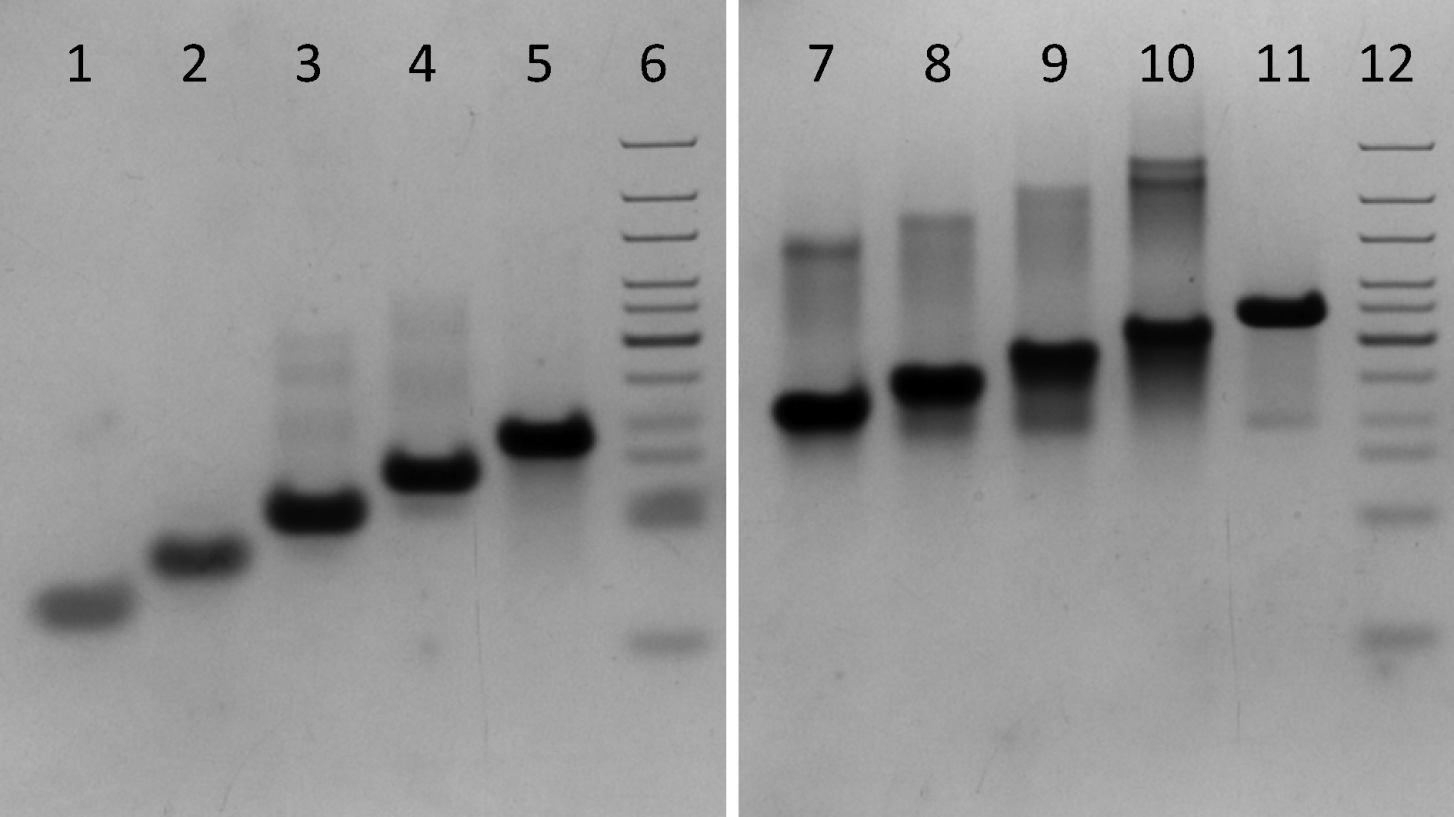
Room temperature native PAGE of the model ODNs (10 μM in 10 mM sodium cacodylate at pH 7.4) with increasing tract lengths at pH 7.4. Acrylamide gels (20%) were buffered with 50 mM tris, 50 mM HEPES at pH 7.4. ODNs were annealed at pH 7.4 as described above. The samples were loaded onto the gel for electrophoresis at 50 V for 5 h. Lane contents left to right are as follows: (1) C_1_T_3_; (2) C_2_T_3_; (3) C_3_T_3_; (4) C_4_T_3_; (5) C_5_T_3_; (6) Base pair ladder standard; (7) C_6_T_3_; (8) C_7_T_3_; (9) C_8_T_3_; (10) C_9_T_3_; (11) C_10_T_3_; (12) Base pair ladder standard.

As the two-stage UV-melting curves suggested that there were two structures assembled from cytosine-rich sequences at pH 7.4, we used native PAGE of all 10 ODNs (C_1-10_T_3_) to determine and visualize the folded species of DNA present. DNA (10 μM) was annealed in 10 mM sodium cacodylate with 100 mM sodium chloride pH 7.4 and allowed to anneal overnight. Samples were then resolved on a 20% acrylamide gel buffered with 50 mM tris, 50 mM HEPES pH 7.4. The first five ODNs with cytosine tracts from 1 to 5 cytosines in length showed a single main species resolved on the gel (Figure 4). This was consistent with the single stage melting curves shown by the C_3-5_T_3_ ODNs. The diffuse bands observed for ODNs C_3-5_T_3_ migrated slower than the main bands and may indicate the presence of intermolecular i-motif species but these were not the dominant form in solution. Species both slower and faster migrating than the primary band were resolved at pH 7.4 for the longer ODNs, C_6-10_T_3_. The slower, and therefore larger species, may be intermolecular i-motifs. Additional bands that migrated further than the primary band can be observed on the gel. This indicated the presence of a species that was folded in such a way that it is more compact and migrated at a different rate than the primary folded species. This supported the presence of a single ODN assuming two folded conformations at pH 7.4, as suggested by the multistage UV melts.

If the TDS data is taken into account, the species resolved on the gel for C_1-2_T_3_ were likely unfolded random coil and the single species resolved for C_3-5_T_3_ were i-motif. The ODNs with cytosine tracts of 6 to 10 nucleobases with multistage melting curves indicating multiple folded DNA species also had multiple species resolved on the native PAGE at pH 7.4 (Figure 4). Considered together, the spectroscopy and native PAGE of longer tract length ODNs indicated that the i-motif was not the only structure present in solution and another species was present. It is possible that the two structures indicated here represented a hairpin and i-motif. Similar structures have been implicated in the regulation of expression through the BCL-2 promoter and the folding kinetics of i-motif secondary structures (18–20,31,32).

**Figure 5.**
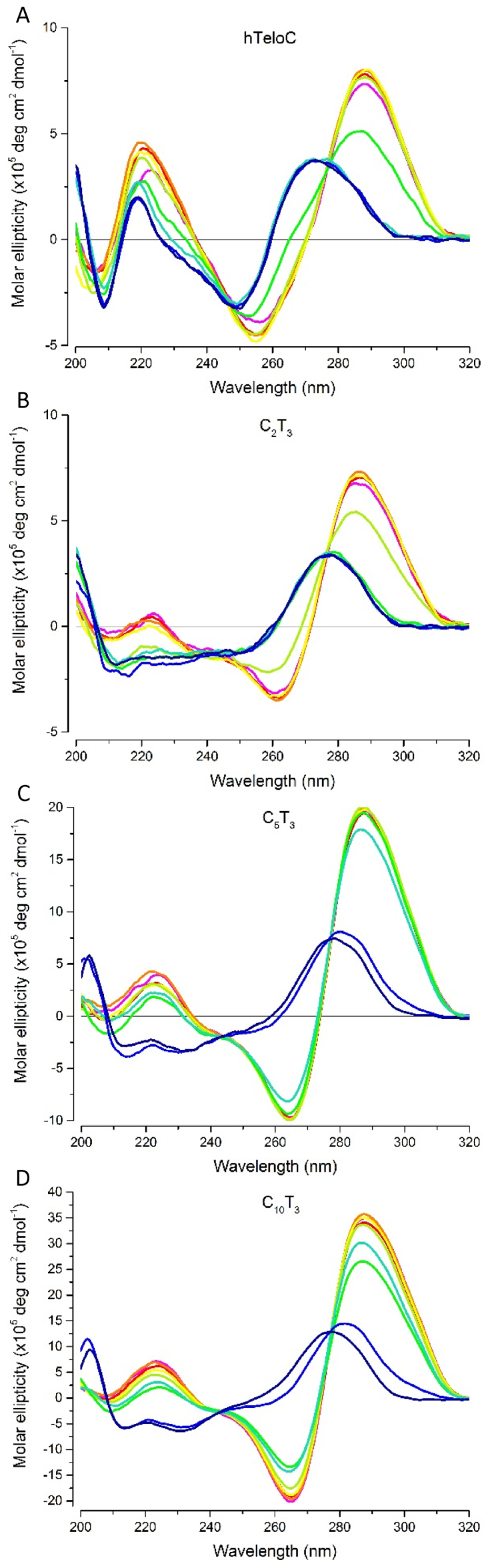
Circular dichroism of (**A**) human telomeric i-motif; (**B**) C_2_T_3_; (**C**) C_5_T_3_ and (**D**) C_10_T_3_. 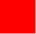 pH 4.0; 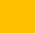 pH 4.5; 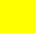 pH 5.0; 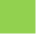 pH 5.5; 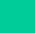 pH 6.0; 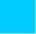 pH 6.5; 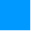 pH 7.0; 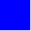 pH 7.5; 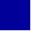 pH 8.0. All ODN concentrations were 10 μM in 10 mM sodium cacodylate buffer with 100 mM sodium chloride buffer at the required pH.

CD was used to further characterize the folded DNA species involved in the melting curves for both experimental pHs. The human telomeric i-motif sequence (hTeloC; Table 1) was included as an example of a previously characterized i-motif. At acidic pH, the folded i-motif conformation was indicated by a positive peak at 288 nm and a negative peak at 260 nm (33); as the pH increases to 6.5, the conformation unfolds, the positive peak shifts to 273 nm and the negative peak to 250 nm (Figure 5A). At pH 5.5, all of the model ODNs showed the positive band at 288 nm that is characteristic of a folded i-motif at acidic pH (Figure 5). As shown previously, C_2_T_3_ represented the minimum cytosine tract requirement for i-motif as C_1_T_3_ did not exhibit the peak at 288 nm (Figure 5B and Supplementary Figure S5A). C_2_T_3_ only displayed the folded conformation spectra until pH 5.5 and unfolded at pH 6.0, demonstrating the typical i-motif preference for acidic environments. C_4_T_3_ showed a spectra consistent with i-motif until pH 6.5 but began to unfold at pH 7.0 as the peak at 288 nm lost amplitude. When the cytosine tract length was increased to five, however, the peaks characteristic of a folded i-motif were present at pH 7.0 and the structure did not unfold until pH 7.5 (Figure 5C). Above tract lengths of five cytosines this stability was not enhanced, but the ODN with six cytosines appeared to be slightly destabilized and the *pH*_T_ fell below pH 7.0, but still near neutral (Table 1). C_10_T_3_ was still stable at a folded conformation up to a pH of 7.0 but unfolded in more alkaline pH environments (Figure 5D). This suggested that i-motif forming sequences with five or more cytosine tracts may fold and remain stable as i-motif at physiological pH.

A brief examination of varied loop lengths with constant tract lengths was also undertaken to determine whether the thermal and pH stability of C_5_T_3_ could be enhanced. Having longer loops between the i-motif cytosine tracts suggested greater folding freedom and possibly reduced likelihood of folding the expected i-motif. It has been suggested that greater levels of flexibility give rise to weaker stabilizing interactions unless the loop regions can contribute to stability with additional base pairing (10). Thymine bases were used for all of the loop nucleobases to keep their contribution to i-motif folding uniform (10). While this simplifies our model and analysis, using loops consisting of only thymine bases may limit how well this data applies to naturally occurring sequences, particularly as these loop regions have been shown to be recognition motifs for protein binding (19,21,34). Maintaining the length of the tract at five cytosines and increasing loop length increased hysteresis (Figure 2B), and indicated slower folding kinetics. Increasing the loop length from 1 to 4 thymines resulted in minimal hysteresis at pH 5.5. At pH 7.4, hysteresis was far more pronounced between C_5_T_1_ and C_5_T_4_, for example, with a 22.3°C difference.

The ODNs with variable loop lengths were also investigated using CD. The ellipticity of each of the ODNs with a constant tract length of five cytosines and variable loop lengths was consistent with folded i-motif at pH 4.0 to 6.0 (Supplementary Figure S5). A loop length of four thymines resulted in a folded conformation with a *pH*_T_ of 6.7 that was only stable at pH 6.5 and unfolded from pH 7.0 to 8.0 (Supplementary Figure S5C). The i-motif with shorter loops (1-3 thymines per loop (Supplementary Figure S5A and B)) remained folded or halfway between the two conformations at pH 7.0 with *pH*_T_s of 6.9, 7.1 and 7.2 for C_5_T_1_, C_5_T_2_ and C_5_T_3_, respectively (Table 1). This suggested that loop length, in addition to sequence (9), contributed to the folding kinetics of i-motif and implied that longer thymine loops were detrimental to i-motif stability (10,11). Thus in the absence of any additional stabilizing interactions within the loop sequence, the optimum loop length was found to be three bases. While loop lengths were kept uniform here, it has been reported that the sequence and length of loops 1 and 3 contribute to i-motif stability at neutral pH (8). This is thought to stem from the contribution of loop bases to hydrogen bonding for greater stability (9). However, longer lengths in loops 1 and 3 produce i-motif that are less stable at higher pH and temperature than those with a longer loop 2 (11). These findings were consistent with previous research, which showed that shorter loop lengths result in higher thermal and pH stability in i-motif (10).

Taken together, the evidence presented here suggested that cytosine tracts of five nucleobases were a tipping point for stability at neutral pH. It has been suggested that the pH in the nucleus (approximately pH 7.3) would preclude i-motif formation (35,36). However, if the basal level stability of i-motif with tracts of five cytosines is higher at physiological pH, the likelihood of i-motif formation in the nucleus of biological systems is far more probable; irrespective of other influences which have been shown to stabilize DNA secondary structure formation such as molecular crowding and negative superhelical tension (14,15). With this in mind, we decided to investigate the potential prevalence of such sequences which meet this criteria within the human genome using Quadparser. Based on the experimental evidence, we developed an i-motif folding rule of the form C_5_(N_1-19_C_5_)_3_ where N can be any base including C (which may give rise to longer tracts). This rule specified a minimum tract length of five cytosines in four tracts with intervening loops of 1 to 19 nucleobases. This was importantly different from the standard G-quadruplex folding rule (G_3+_N_1-7_)_3_G_3+_). The requirement for four runs of five cytosines made it relatively much rarer, although the more relaxed loop length requirements alleviated this somewhat and accounted for potential loop to loop variation in natural i-motif sequences. A total of 5125 sequences with i-motif potential were identified from the genome. We investigated the effect of loop length constraints on the number of i-motifs identified, and found that the number varied roughly linearly with the maximum loop length (in the range 7–19). In contrast, simple probability would predict that the number should vary with the cube of the loop length.

We then investigated whether the sequences identified were randomly located or localized immediately upstream of transcription start sites. We found that 637 of the predicted stable i-motif structures overlap gene promoters (defined here as the 1 kb region upstream of a known gene). This represented 12.4% of all the identified sequences, significantly larger than the 2.4% predicted by chance (p << 0.05, chi squared ∼370). They split almost equally as to which of the two strands could form the i-motif. Noticeably, 30 genes have multiple i-motif sequences identified in their promoter regions, including two Shank genes (full table in Supplementary Data).

It is known that G-quadruplex forming sequences are preferentially located in the promoters of certain types of genes, and so we sought to investigate whether this was true for i-motif sequences as well. Because there are many fewer predicted i-motifs, we examined all gene ontology codes applicable to at least 100 genes. We then used the Benjamini and Hochberg technique to reduce false discovery rates resulting from multiple hypothesis testing, and even under these stringent conditions found a number of gene types significantly more or less likely to contain i-motifs. Genes involved in skeletal system development, sequence specific DNA binding, DNA templated transcription and positive regulation of transcription from RNA polymerase II promoter were significantly more likely to have i-motifs, whereas genes involved in the immune response, G-protein coupled receptor activity and olfactory receptor activity were significantly unlikely to contain i-motifs.

We selected 33 example putative genomic i-motif forming sequences for further characterization with UV spectroscopy, TDS and CD. The sequences were from different positions, strands (coding/non-coding), chromosomes and varied in length between 23 and 116 nucleotides. Of the 33 genomic ODNs selected, 17 were found to have a *pH*_T_ ≥ 7.0 although none of these sequences had a higher *pH*_T_ than C_5_T_3_ (Table 2). However, 7 of the 33 showed evidence of an i-motif (indicated by TDS (Supplementary Figure S11)) with a higher *T*_m_ than the model sequence. An additional 12 sequences were found to have a *T*_m_ ≥ 20°C (Supplementary Figure S12). This evidence indicates that there are multiple stable i-motif forming sequences in the genome. However, this is by no means a comprehensive examination of all the potential i-motif forming sequences in the human genome. Given the complexity of the i-motif structure, confidently predicting and identifying sequences that form natural i-motif remains challenging. Nevertheless, these findings present a starting point to identify potential regions for further study.

**Figure 6.**
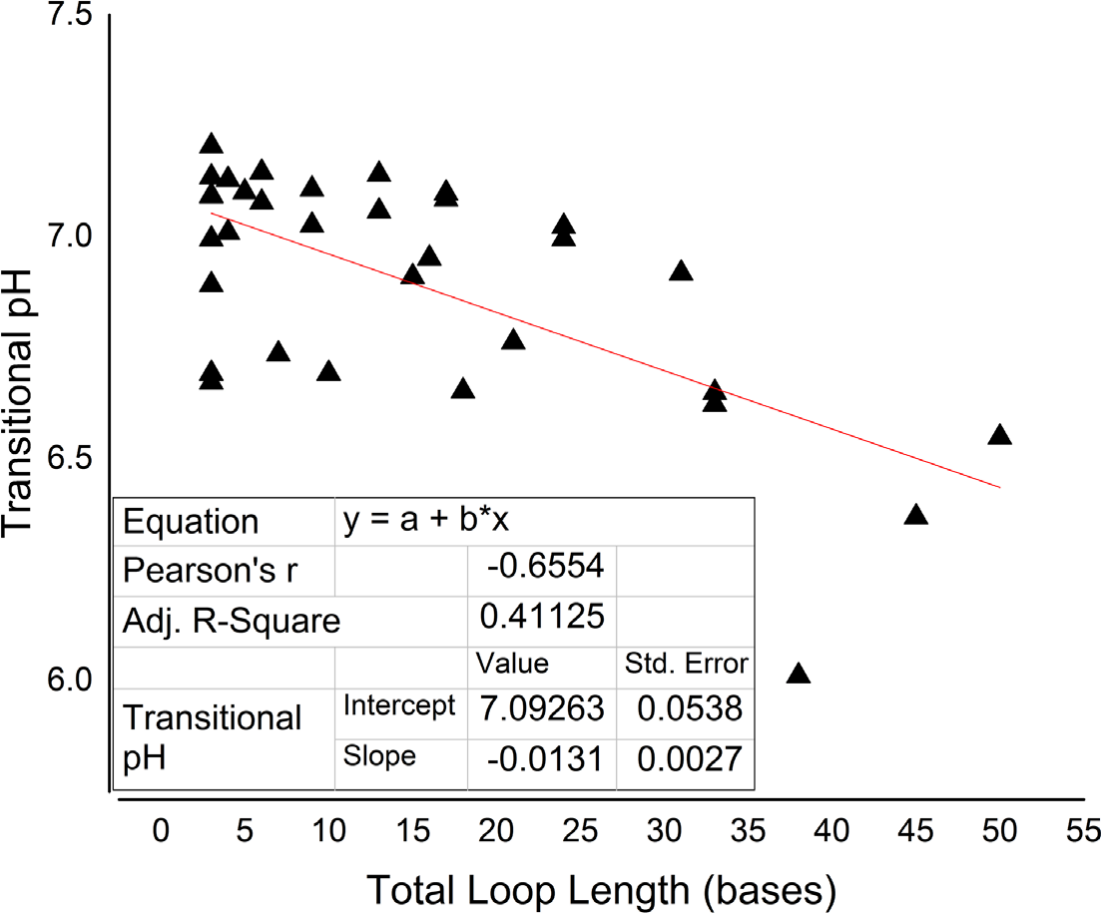
The relationship between total loop length (the sum of all loop bases) and transitional pH. Transitional pH was calculated from fitting the CD data for all pH at 288 nm and identifying the inflection point.

**Figure 7.**
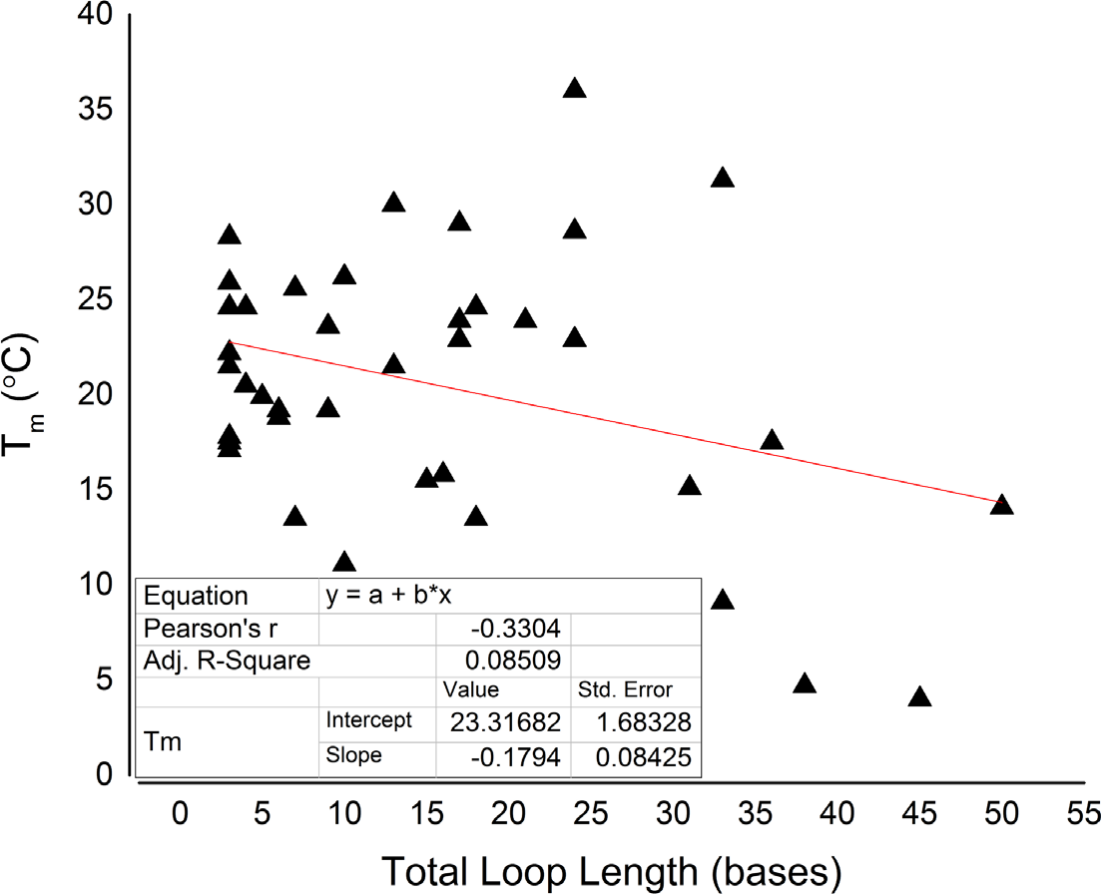
The relationship between total loop length (the sum of all loop bases) and melting temperature (*T*_m_). *T*_m_ was calculated from the maxima of the first derivative of UV-melt data.

We examined the sequence characteristics of the genomic i-motif candidates to see whether tract length, loop length and number of loops or tracts showed any correlation with pH or thermal stability. When examined in relation to total loop length, a moderate negative correlation (*r* = −0.65548) between *pH*_T_ and total loop length was observed (Figure 6). A similar trend was observed for melting temperature and total loop length (Figure 7), although with only a weak negative correlation (*r* = −0.3304). This showed that in human genomic candidate i-motif sequences, both transitional pH and melting temperature decrease with increasing total loop length, although clearly other factors, such as the precise sequence of the loops, play a role. This supports our finding that model i-motif with shorter loops are inherently more stable. This trend can also be observed in genomic cytosine-rich sequences which have more variability than model sequences.

## CONCLUSION

Despite the many potential i-motif forming sequences found in the genome and at high concentration in promoter regions and regulatory genes, the pH of physiological conditions remains one of the main obstacles for i-motif formation *in vivo*. While *in vivo* evidence for i-motif formation is still yet to be observed, we have demonstrated that neutral pH does not exclude i-motif formation. The work presented here clearly supports this statement and shows that it is possible to achieve i-motif stability at physiological pH without the use of crowding agents, provided there is a minimum tract length of five cytosines. This investigation allowed for the development of an i-motif folding rule that identified candidate cytosine-rich sequences. This preliminary search for stable i-motif was not comprehensive but gave a starting indication of potential i-motif forming sequences in the human genome comprising sequence characteristics that make neutral folding possible.

Over half of the genomic ODNs tested here have shown evidence of an i-motif at neutral pH. Not only does this highlight 17 new genomic i-motif candidates but also shows the potential for i-motif in biological systems. The location of these potential i-motif forming sequences throughout the genome suggests a widespread prospective field of influence on transcription for these structures. We further demonstrated that these sequences are not randomly located, but are found predominantly in gene promoters of specific gene types. This shows that these sequences, which have the capacity to fold under neutral conditions are also located where their folding can have a physiological effect. This has enormous potential for identifying physiological i-motif and probing their function in biological systems.

## Supplementary Material

Supplementary Data
