# Identification of multiple genomic DNA sequences which form i-motif structures at neutral pH

**DOI:** 10.1093/nar/gkx1178

**Published:** 2017-11-13

**Authors:** Elisé P. Wright, Julian L. Huppert, Zöe A.E. Waller

**Affiliations:** 1 School of Pharmacy, University of East Anglia, Norwich Research Park, Norwich NR4 7TJ, UK; 2 Intellectual Forum, Jesus College, University of Cambridge, Cambridge CB5 8BL, UK; 3 Centre for Molecular and Structural Biochemistry, University of East Anglia, Norwich Research Park, Norwich NR4 7TJ, UK

*Nucleic Acids Research* (2017) 45(6) 2951–2959, https://doi.org/10.1093/nar/gkx090

The authors wish to make the following correction to their article:

The last column of Table 2, pH_T_, is not in the correct order. A new table is provided below and the table has been corrected in the published article.

**Table 2.** Thermal and pH stability of the genomic i-motif candidate oligonucleotides used in this study

			pH 7.0		
Notation	Bases	Sequence 5′ - 3′	*T*_m1_/*T*_m2_	*T*_a_	*pH_T_*
AC017019.1	28	CCC-CCC-TCC-CCC-CCT-CCC-CCC-TCC-CCC-C	27.9 ± 0.6	18.0 ± 0.2	7.1
AC018878.3	26	CCC-CCA-CCC-CCA-GCC-CCC-TTT-CCC-CC	18.1 ± 0.2	7.5 ± 0.5	7.1
ATXN2L	24	CCC-CCC-CCC-CCC-CCC-CCC-CCC-CCC	23.7 ± 1.0	22.5 ± 0.6	7.0
CAMK2G	50	CCC-CCA-GGC-CCC-GCC-AGT-CCC-CCC-CCC-CGC-CCG-GCC-CCC-GGC-CCG-CCC-CC	13.0 ± 1.2	11.3 ± 0.7	6.9
DAP	29	CCC-CCG-CCC-CCG-CCC-CCG-CCC-CCG-CCC-CC	24.7 ± 0.5	22.0 ± 0.4	7.0
DRP2	70	CCC-CCT-CTT-CCC-CTC-TCC-CCC-TCT-CCC-CCT-CTC-TCC-CTC-TTC-CCC-CTC-TCC-TTG-TCT-CCTTCT-CTC-CCC-C	4.5 ± 0.7	6.5 ± 0.7	6.0
DUX4L22	41	CCC-CCG-AAA-CGC-GCC-CCC-CTC-CCC-CCT-CCC-CCC-TCT-CCC-CC	29.2 ± 0.2	14.2 ± 0.2	7.1
GH2	42	CCC-CCA-CCC-CCA-CCC-CCA-TCC-CCA-CGC-CCC-GCC-CCC-GCC-CCC	22.7 ± 1.0	15.2 ± 0.9	7.1
HIC2	74	CCC-CCG-GGA-CAG-GGA-CCC-TGG-CCC-CCC-CCG-ACA-GGC-TGA-CGC-CCA-CCC-CCT-CAA-ACT-CTG-GTG-GAC-TTA-CCC-CC	7.5 ± 2.1	7.8 ± 2.0	6.4
HOXC10	24	CCC-CCA-CCC-CCA-CCC-CCA-CCC-CCC	17.7 ± 0.8	14.0 ± 0.2	7.1
HOXD10	26	CCC-CCC-CCC-CCT-CCC-CCG-CGG-CCC-CC	10.2 ± 0.9	5.2 ± 0.2	7.1
JAZF1	31	CCC-CCC-CCG-CCC-CCG-CCC-CCG-CCC-TCC-CCC-C	20.4 ± 0.4	18.5 ± 0.6	7.1
MSMO1	23	CCC-CCG-CCC-CCG-CCC-CCG-CCC-CC	16.6 ± 0.5	15.9 ± 0.4	6.7
NFATC1	45	CCC-CCG-TTT-CCC-CCG-CCA-GCC-CCA-GCG-CCC-CCC-TGC-CCG-GCC-CCC	23.2 ± 0.0/28.8 ± 0.5	18.8 ± 0.6	7.1
PIM1	45	CCC-CCG-ACG-CGC-CCC-CCA-ACA-CAC-AAA-CCC-CCA-GAA-TCC-GCC-CCC	29.4 ± 1.9	5.1 ± 0.2	7.0
PLCB2	36	CCC-CCG-CCT-CTT-CTG-GAG-GCC-CCC-GCC-CCC-ACC-CCC	15.0 ± 0.2	13.2 ± 0.2	7.0
QSOX1	25	CCC-CCG-CCC-CCG-AGC-CCC-CGC-CCC-C	20.1 ± 0.2	11.9 ± 0.4	7.1
RAE1	116	CCC-CCC-GCC-CCC-CCC-GCC-CCC-CCG-CGC-CGC-CCC-CCC-CCG-CCC-CCC-GCC-CCC-GTC-CCC-CCG-CCC-CCC-CCG-CCC-CCC-CCG-CCC-CCC-GTC-CCC-CCG-CCC-CCC-CGC-CCC-CCC-GTC-CCC-CC	27.1 ± 7.4	13.6 ± 6.3	6.8
RUNX1–1	31	CCC-CCC-CCG-CAC-CCC-TTC-CCC-CGG-CCC-CCC-C	14.3 ± 0.9/25.0 ± 0.4	9.8 ± 0.4	6.7
RUNX1–2	36	CCC-CCC-TCC-CCC-TGC-CTC-TCC-CTC-CCC-CCT-TTC-CCC	13.2 ± 0.2/24.4 ± 0.2	10.1 ± 0.0	6.5
RUNX1–3	32	CCC-CCC-TTT-CCC-CTG-CCC-CCC-CTG-CCT-CCC-CC	10.7 ± 0.6/26.2 ± 0.0	9.7 ± 0.6	6.7
SHANK1b	28	CCC-CCC-TCC-CCC-CAC-CCC-CCA-CCC-CCC-C	22.5 ± 0.6	12.0 ± 0.0	7.1
SHANK3	80	CCC-CCG-CCT-CCG-GCG-CAG-CCC-CCT-CGC-CAC-CCC-CGC-TTC-CCT-CCC-GTC-TCA-GGC-CCC-CTC-CCC-CCG-CCG-CCC-CCG-CCC-CC	18.3 ± 1.9	5.6 ± 1.0	6.6
SHANK3b	79	CCC-CCC-GCA-CCG-AGG-CCT-AGG-ACT-CCC-CCC-CCC-AAC-CCC-GTC-ACA-GCC-CCC-CAG-ACC-CCC-GCC-CCG-TGG-CTC-GGC-CCC-C	12.6 ± 3.6	4.8 ± 0.7	6.5
SNORD112	36	CCC-CCC-CCC-GCC-CCC-CAC-CCC-CCC-ACC-CCC-CCC-CCC	25.8 ± 0.5	15.9 ± 0.4	7.2
SOX1	57	CCC-CCT-GCA-GGC-CCC-CCT-GCG-CCT-CCC-CCC-CCC-CGC-CAC-TGG-CGC-CTG-GCT-TCC-CCC	9.0 ± 0.2	6.5 ± 0.5	6.9
STX17	33	CCC-CCG-CCC-CCG-CCC-CCG-CCC-CGC-AGG-GCC-CCC	19.5 ± 0.6	15.0 ± 0.2	7.0
Tandem Repeat (LA16c-OS12.2)	57	CCC-CCC-GTG-TCG-CTG-TTC-CCC-CCG-TGT-CGC-TGT-TCC-CCC-CGT-GTC-GCT-GTT-CCC-CCC	9.4 ± 1.5/31.6 ± 0.6	6.7 ± 0.6	6.6
TRABD	23	CCC-CCG-CCC-CCC-CCC-CCC-CCC-CC	21.3 ± 0.2	19.3 ± 0.2	6.9
WNT7A	48	CCC-CCG-CCC-CTC-CCT-CCT-TTC-CCC-CGT-CCC-TCC-CCC-GCC-CCC-TCC-CCC	22.7 ± 2.5	16.1 ± 0.0	7.1
ZBTB7B	58	CCC-CCC-ATC-CCT-CCC-CTC-CCT-CCC-CCC-GCC-CCT-GCC-ACC-CCC-CAA-ACT-CCC-CCC-CCC-C	25.5 ± 1.3	10.0 ± 0.2	7.1
ZFP41	52	CCC-CCA-GCC-CCC-GCC-GAC-CCC-CAG-CTC-CCG-CCT-CCG-CCG-ACC-CCC-AGC-CCC-C	21.7 ± 0.7/35.6 ± 0.4	17.4 ± 0.4	7.0
ZNF480	23	CCC-CCG-CCC-CCG-CCC-CCG-CCC-CC	17.1 ± 0.0	15.2 ± 0.2	6.7

The mistake does not affect the overall conclusions of the work.

