# Supplementary material for: Identification of multiple genomic DNA sequences which form i-motif structures at neutral pH
Source: Nucleic Acids Res. 2017 Feb 9;45(6):2951–9. doi: 10.1093/nar/gkx090 (PMC5605235; doi:10.1093/nar/gkx090)
Supplement: Supplementary Data [file gkx090_Supp.pdf]

SUPPLEMENTARY INFORMATION

Identification of multiple genomic DNA sequences which form i-motif structures at neutral pH

Elisé P. Wright[1], Julian L. Huppert[2] and Zoë A. E. Waller[1,3]*

[1.] School of Pharmacy, University of East Anglia, Norwich Research Park, Norwich, NR4 7TJ
[2.] Intellectual Forum, Jesus College, University of Cambridge CB5 8BL.
[3.] Centre for Molecular and Structural Biochemistry, University of East Anglia, Norwich Research Park, Norwich, NR4 7TJ.

* To whom correspondence should be addressed. Tel: +44 (0) 1603 59 1972; Fax: +44 (0) 1603 59 2003; Email: z.waller@uea.ac.uk.

Present Address: Z. A. E. Waller, School of Pharmacy, University of East Anglia, Norwich Research Park, Norwich, NR4 7TJ, UK.

Contents

**1.**	**Model Library**	**S1**
**2.**	**Control Sequences**	**S8**
**3.**	**Genome Searching using Quadparser**	**S11**
**4.**	**Genomic i-motif candidate sequences**	**S12**

1. MODEL LIBRARY

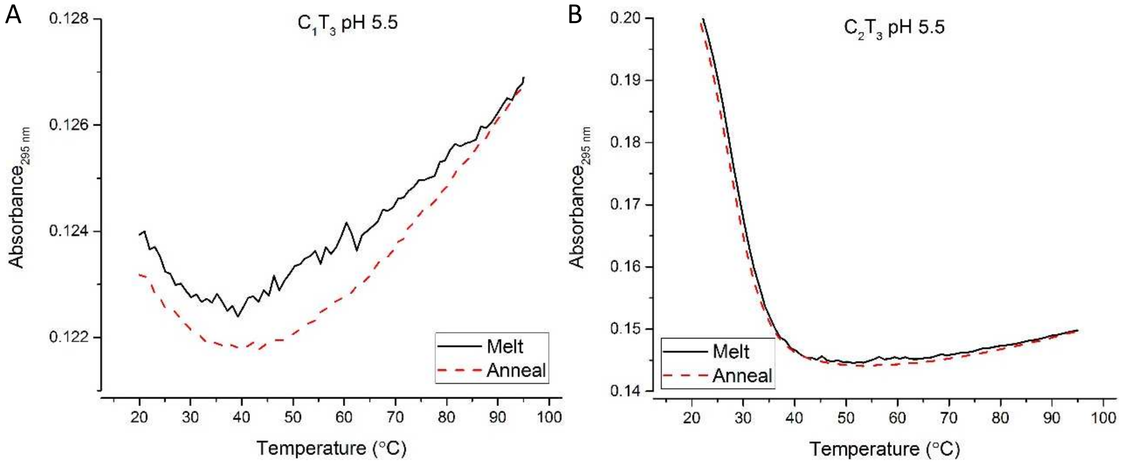

Figure S1. The UV melting and annealing curves for each of the model ODNs (2.5 μM) which were annealed in 10 mM sodium cacodylate with 100 mM sodium chloride at pH 5.5.

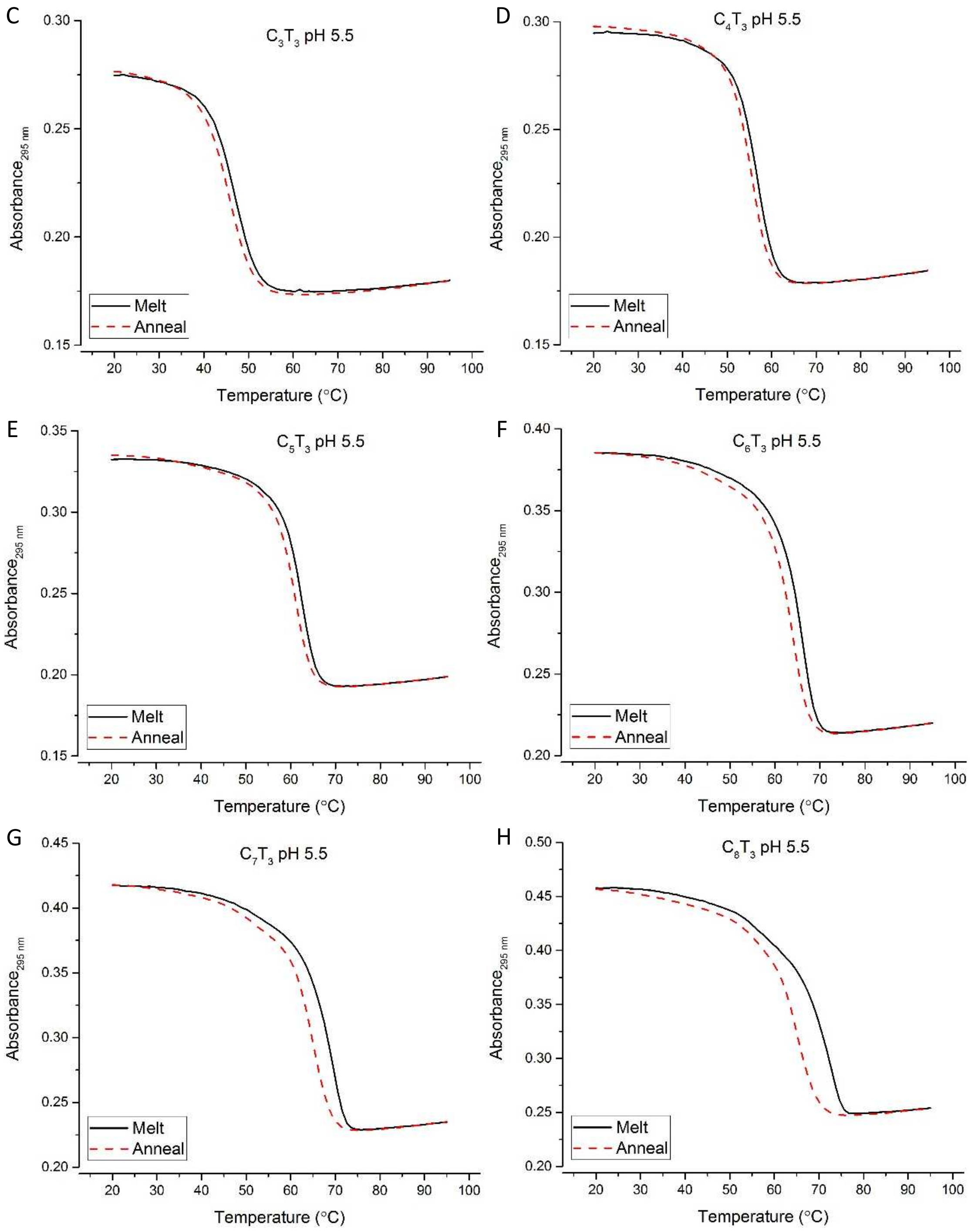

Figure S1. The UV melting and annealing curves for each of the model ODNs (2.5 μM) which were annealed in 10 mM sodium cacodylate with 100 mM sodium chloride at pH 5.5.

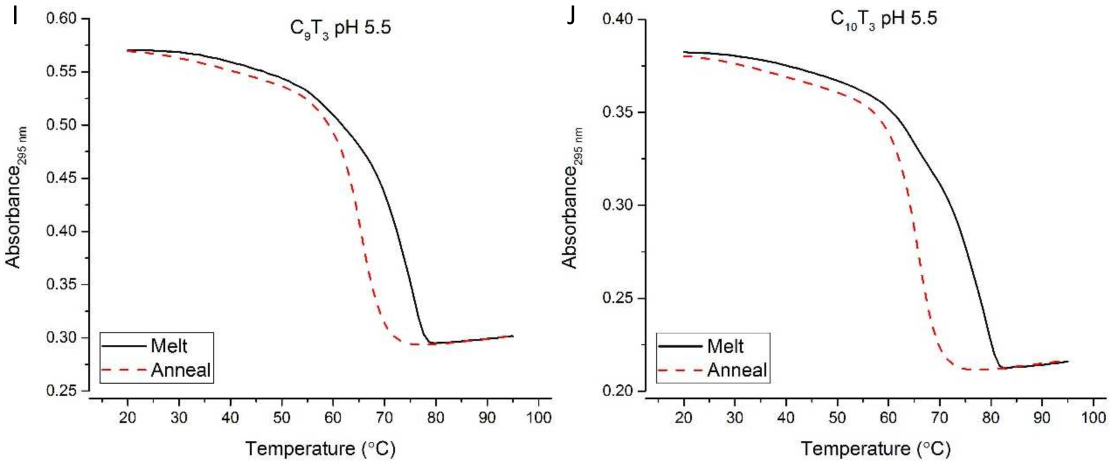

Figure S1. The UV melting and annealing curves for each of the model ODNs (2.5 µM) which were annealed in 10 mM sodium cacodylate with 100 mM sodium chloride at pH 5.5.

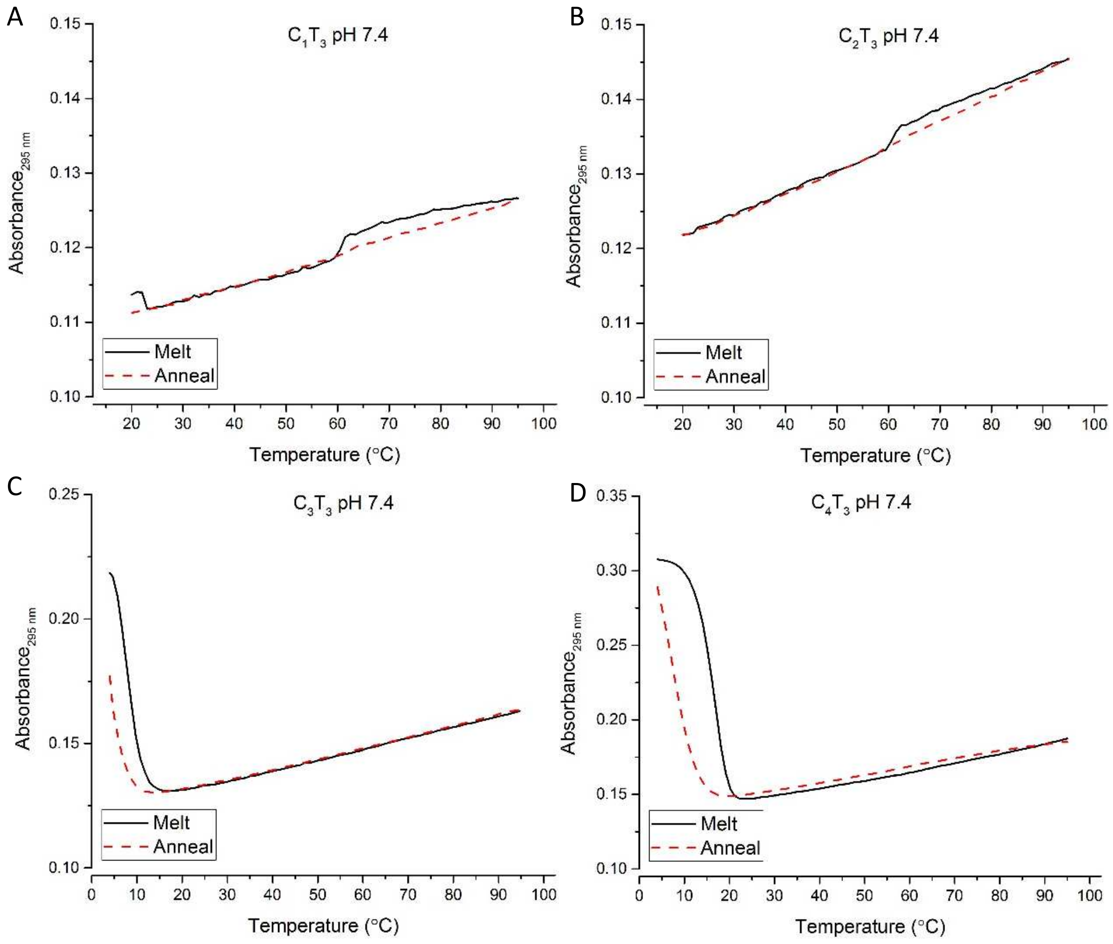

Figure S2. The UV melting and annealing curves for each of the model ODNs (2.5 µM) which were annealed in 10 mM sodium cacodylate with 100 mM sodium chloride at pH 7.4.

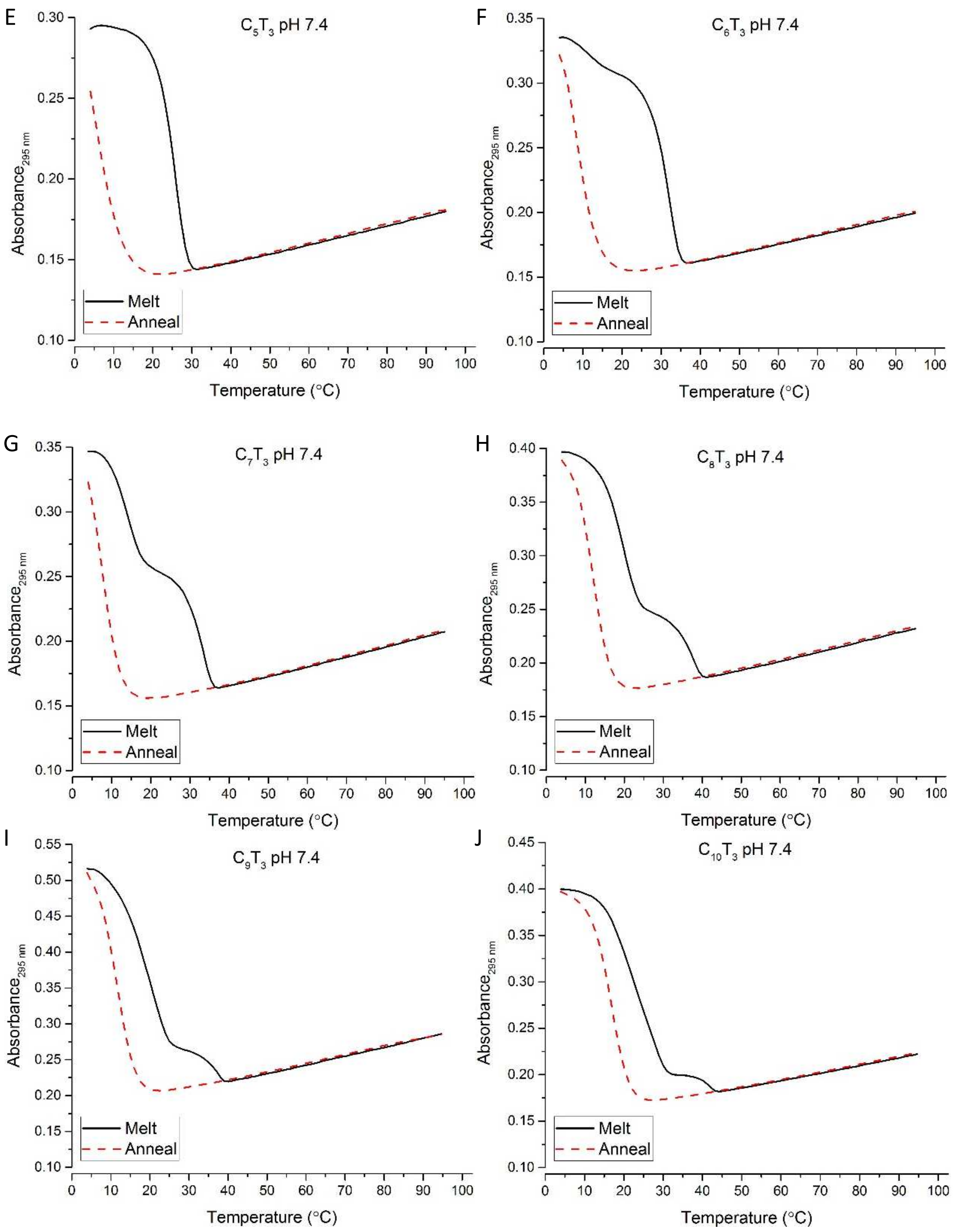

Figure S2. The UV melting and annealing curves for each of the model ODNs (2.5 µM) which were annealed in 10 mM sodium cacodylate with 100 mM sodium chloride at pH 7.4.

Additional UV melting experiments were undertaken to examine whether the multistage UV melts at pH 7.4 were concentration dependent. UV melt experiments using C_7T_3 at concentrations of 1.25 and 5 µM were carried out using the same experimental parameters as previously described. The results show that even at both half and double the concentration, the shape of the multistage melting curve is consistent (Figure S3). This provides evidence that the multistage melts do not arise as a result of intermolecular oligonucleotide interactions.

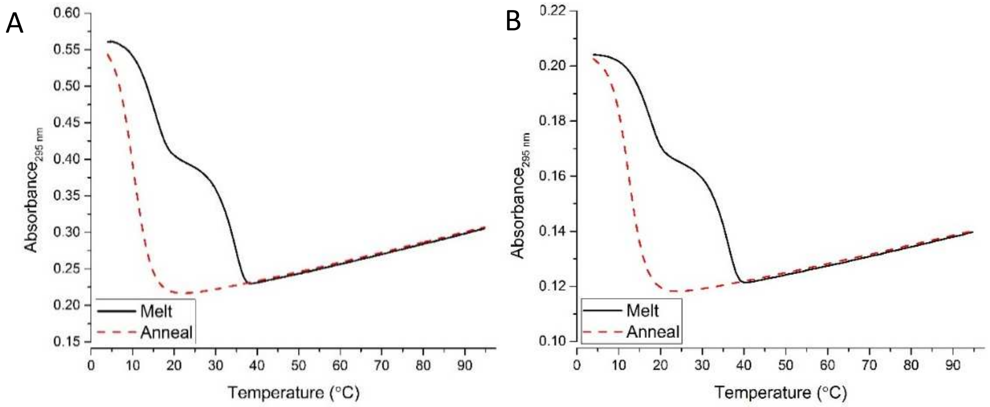

Figure S3. UV melting experiments of C7TTT at 1.25 µM (A) and 5 µM (B). ODNs were annealed in 10 mM sodium cacodylate with 100 mM sodium chloride at pH 7.4

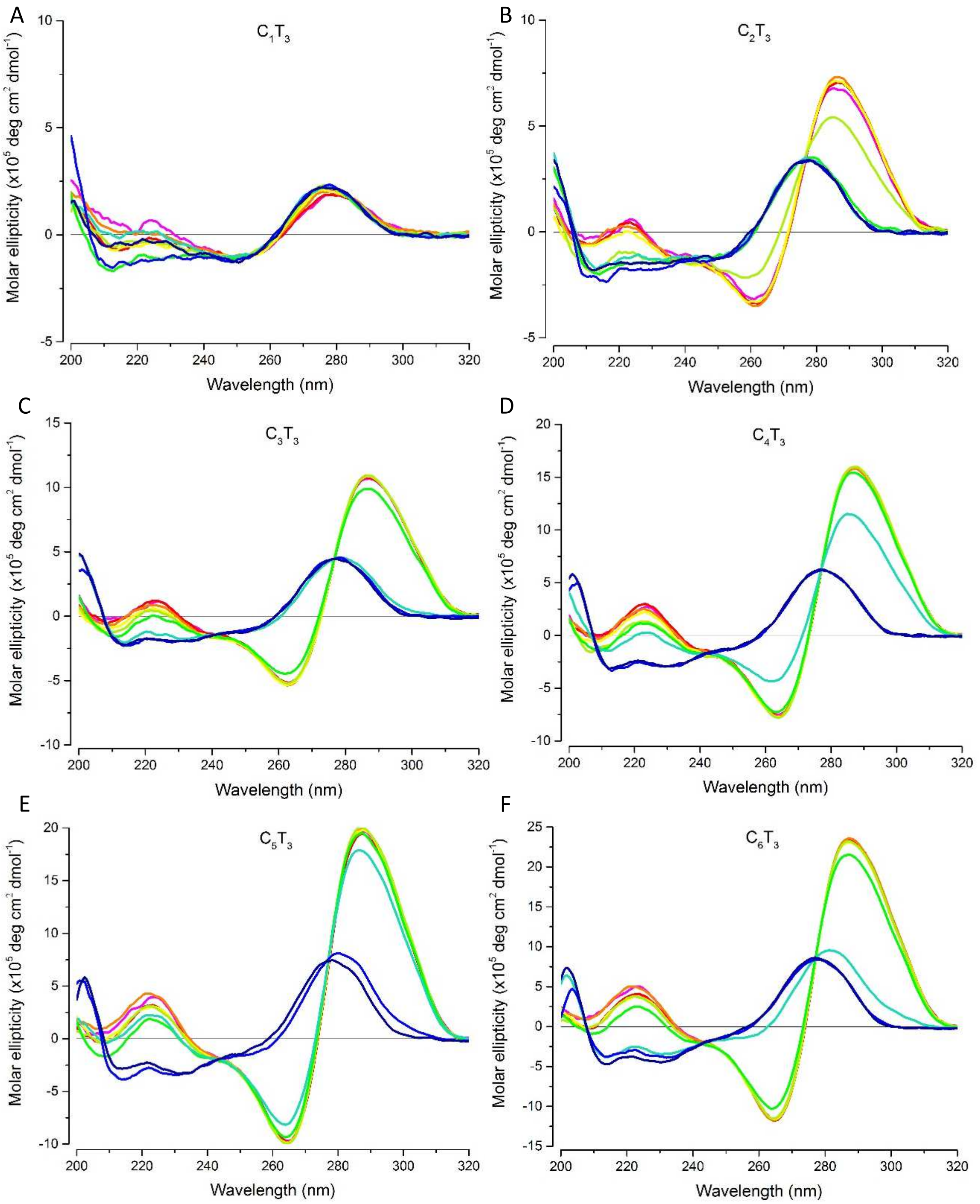

Figure S4. The circular dichroism of each of the model ODNs (10 μM) measured in 10 mM sodium cacodylate with 100 mM sodium chloride at pH 4.0 to 8.0 in 0.5 pH unit increments. All signals were buffer subtracted. A) C_1T_3; B) C_2T_3 ; C) C_3T_3; D) C_4T_3; E) C_5T_3; and F) C_6T_3. ■pH 4.0; ■pH 4.5; ■pH 5.0; ■pH 5.5; ■pH 6.0; ■pH 6.5; ■pH 7.0; ■pH 7.5; ■pH 8.0.

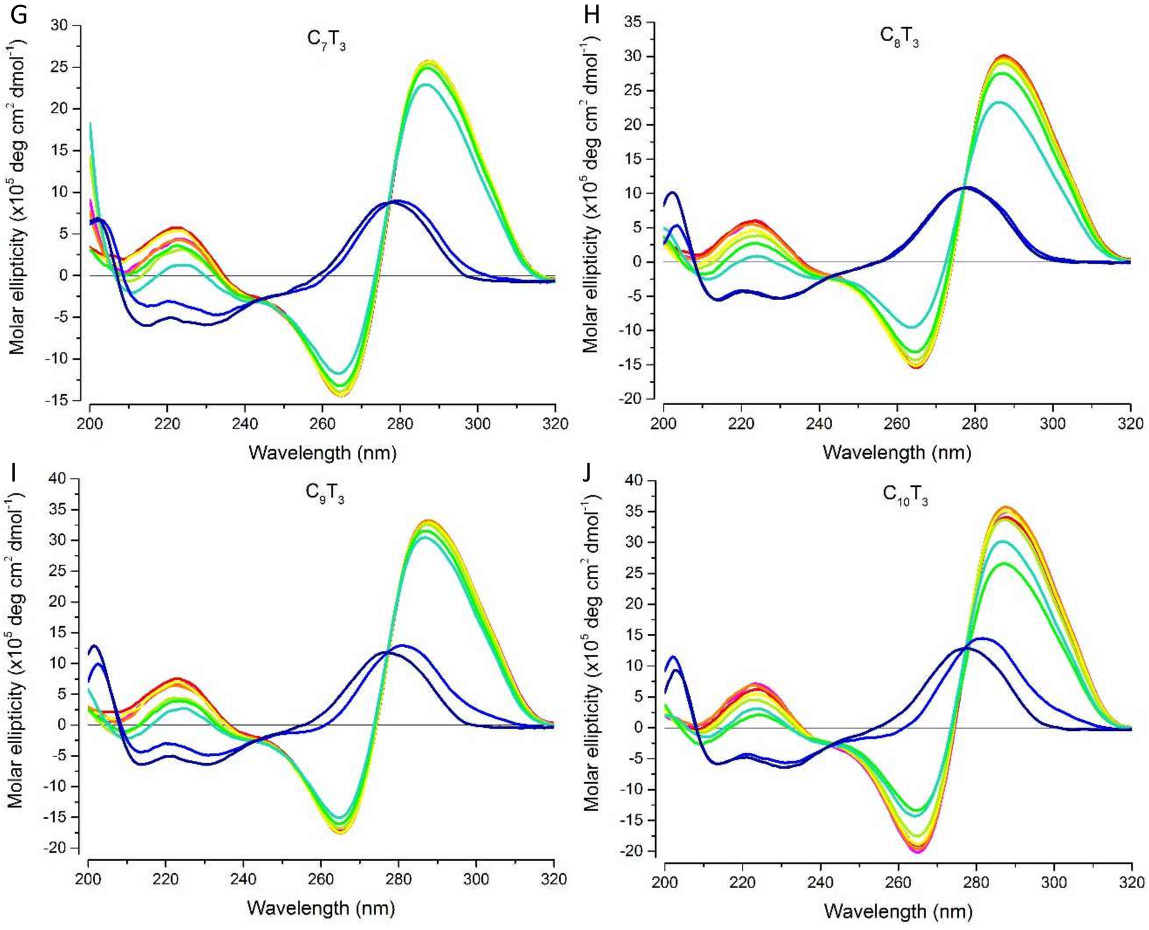

Figure S4. The circular dichroism of each of the model ODNs (10 µM) measured in 10 mM sodium cacodylate with 100 mM sodium chloride at pH 4.0 to 8.0 in 0.5 pH unit increments. All signals were buffer subtracted. G) C_7T_3; H) C_8T_3; I) C_9T_3; and J) $C_{10}T_3$. ■pH 4.0; ■pH 4.5; ■pH 5.0; ■pH 5.5; ■pH 6.0; ■pH 6.5; ■pH 7.0; ■pH 7.5; ■pH 8.0.

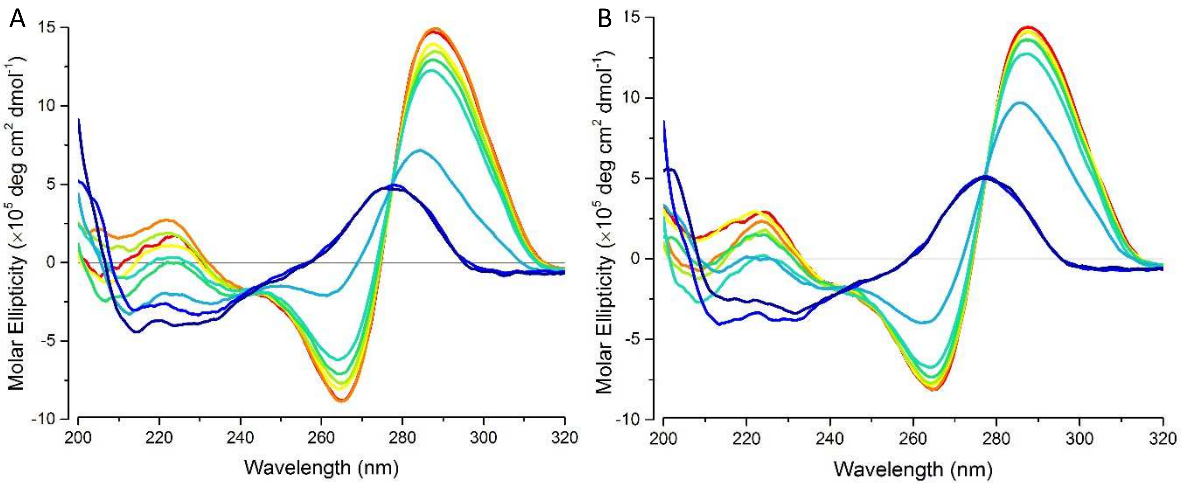

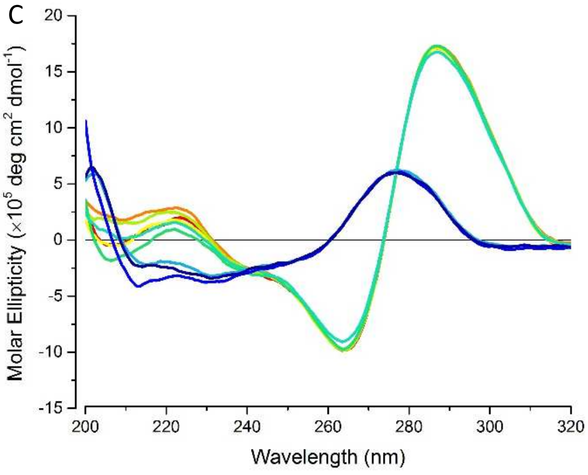

Figure S5. The circular dichroism of each of the variable loop length ODNs (10 µM) measured in 10 mM sodium cacodylate with 100 mM sodium chloride at pH 4.0 to 8.0 in 0.5 pH unit increments. All signals were buffer subtracted. A) C_5T_1; B) C_5T_2; and C) C_5T_4. ■pH 4.0; ■pH 4.5; ■pH 5.0; ■pH 5.5; ■pH 6.0; ■pH 6.5; ■pH 7.0; ■pH 7.5; ■pH 8.0.

2. CONTROL SEQUENCES

Our study showed that C_5T_3 is stable at room temperature and neutral pH. To test whether the stability arises from the length of the tract, as opposed to the length of the sequence, additional C-rich sequences which were the same length, but had shorter cytosine tracts (1-3 cytosines) were examined. These sequences (UnifC_1T_3, UnifC_2T_3, and UnifC3T3, Table S1) were used to determine whether sequences with shorter tracts that were the same length as C_5T_3 would form similarly stable i-motif. Additionally, a scrambled sequence (ScrC_5T_3, Table S1) was used to determine whether a sequence with the same cytosine content but without the four tract/three loop arrangement would be capable of folding a secondary structure of similar stability to C_5T_3. The melting temperatures of these sequences at pH 5.5 and 7.4 show that a scrambled cytosine content does not give rise to a secondary structure that is comparable to that of folded C_5T_3. In an acidic environment, the stability of the sequences increases with tract length. The similarity between UnifC_2T_3 and ScrC_5T_3 is likely due to coincidental tracts of two cytosines distributed throughout the scrambled sequence. At pH 7.4, UnifC_3T_3 has the highest

melting point at 20.9°C but this structure does not have the pH stability of C_5T_3 (Figure S4). The stability of C_5T_3 is specific to the four tract/three loop arrangement with tract lengths of five cytosines.

Table S1. The T_m for C_5T_3 and control C-rich sequences at pH 5.5 and 7.4.

**Notation**	**Sequence 5' - 3'**	**bases**	**pH 5.5**		**pH 7.4**		**pH_T**
			**T_m** (°C)	**T_a** (°C)	**T_m** (°C)	**T_a** (°C)	
C_5T_3	$C_5(T_3C_7)_5$	29	61.4 ± 0.6	60.6 ± 0.0	26.2 ± 1.8	6.7 ± 0.6	7.2
UnifC_1T_3	$C(T_3C_1)_7$	29	4.0 ± 0.0	5.0 ± 0.0	4.7 ± 0.6	5.0 ± 0.0	ND
UnifC_2T_3	$T(C_2T_3)_5CCT$	29	32.5 ± 0.4	31.6 ± 0.6	5.0 ± 0.0	5.7 ± 0.6	6.2
UnifC_3T_3	$C(T_3C_3)_4TTTC$	29	45.7 ± 0.4	44.4 ± 0.0	19.7 ± 0.8	6.0 ± 1.0	6.7
ScrC_5T_3	CCC-TCC-CTC-TCT-CCT-CTC-CCT-CCT-CCT-CC	29	45.1 ± 2.1	44.4 ± 0.0	9.6 ± 0.8	6.4 ± 0.5	6.6

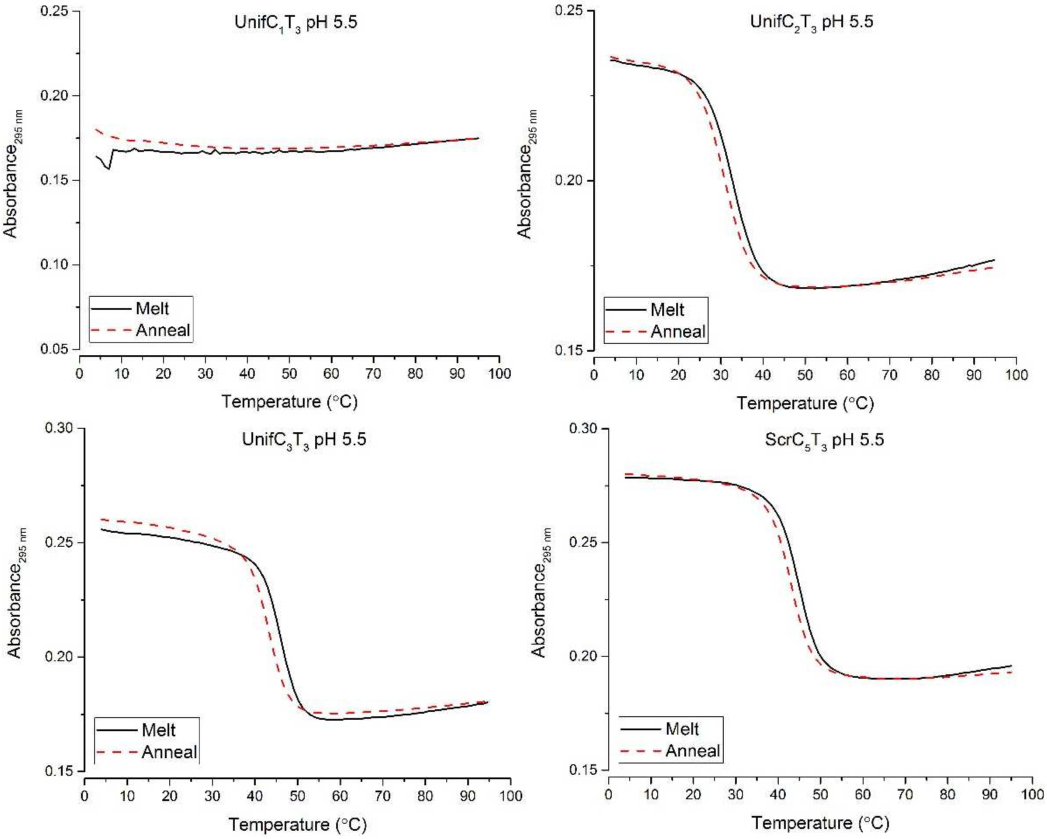

Figure S6. The UV melting and annealing curves for each of the control sequences (Table S1) at 2.5 µM in 10 mM sodium cacodylate with 100 mM sodium chloride at pH 5.5.

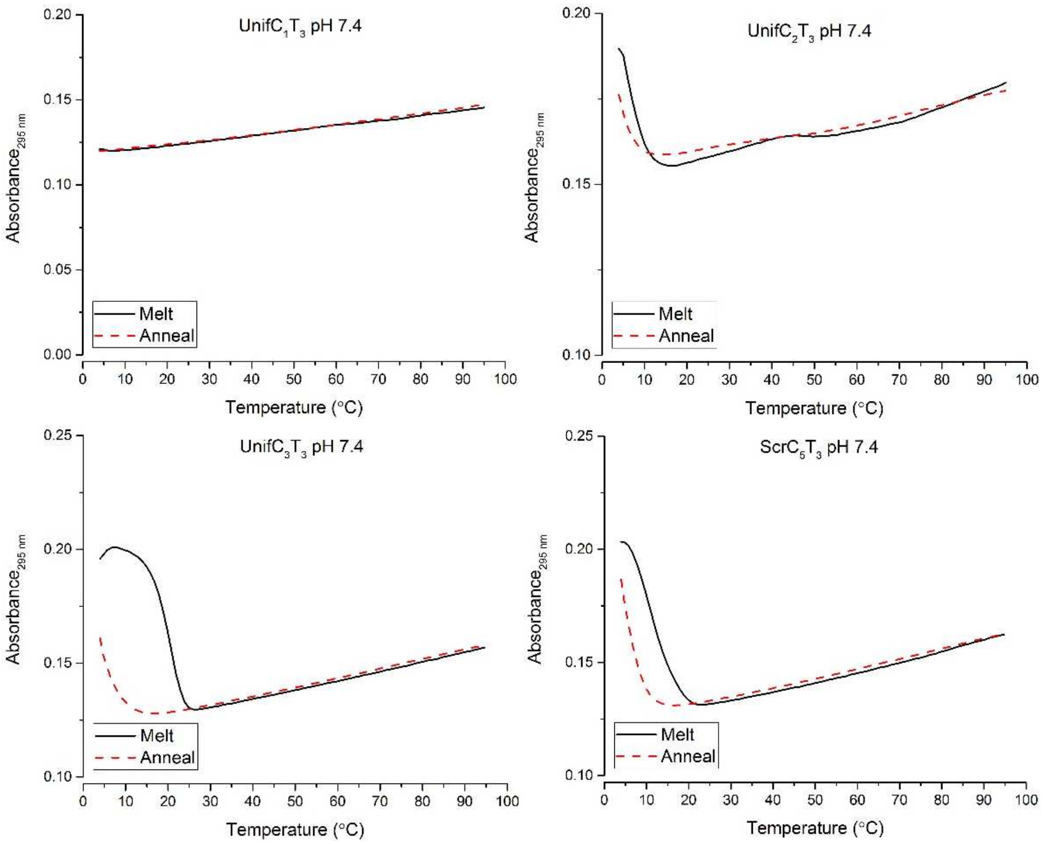

Figure S7. The UV melting and annealing curves for each of the control sequences (Table S1) at 2.5 µM in 10 mM sodium cacodylate with 100 mM sodium chloride at pH 7.4..

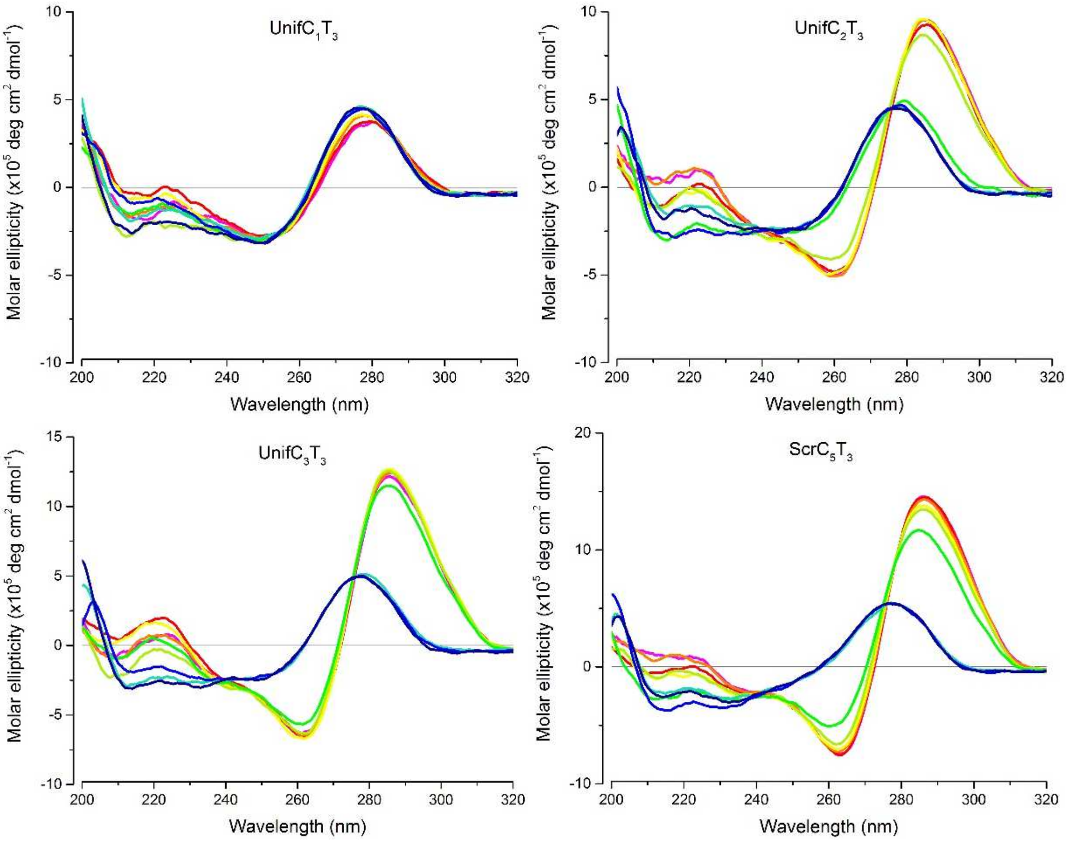

Figure S8. The circular dichroism of the control sequences (10 µM) (Table S1) measured in 10 mM sodium cacodylate with 100 mM sodium chloride at pH 4.0 to 8.0 in 0.5 pH unit increments. All signals were buffer subtracted. ■pH 4.0; ■pH 4.5; ■pH 5.0; ■pH 5.5; ■pH 6.0; ■pH 6.5; ■pH 7.0; ■pH 7.5; ■pH 8.0.

3. GENOME SEARCHING USING QUADPARSER

Table S2. Number of potential i-motif forming sequences in human gene promoters by chromosome.

Chromosome	Number of putative i-motif forming sequences
Y	3
X	29
22	29
21	18
20	20
19	51
18	5
17	47
16	36
15	13
14	14
13	11
12	25
11	32
10	41
9	21
8	19
7	32
6	24
5	16
4	28
3	27
2	42
1	54
**Total**	**637**

4. GENOMIC I-MOTIF CANDIDATE SEQUENCES

Table S3. 33 i-motif candidate sequences selected at random for further secondary structure characterisation shown here in alphabetical order.

Name	Sequence	Chr	Length
AC017019.1	CCC-CCC-TCC-CCC-CCT-CCC-CCC-TCC-CCC-C	Y	28
AC018878.3	CCC-CCA-CCC-CCA-GCC-CCC-TTT-CCC-CC	2	26
ATXN2L	CCC-CCC-CCC-CCC-CCC-CCC-CCC-CCC	16	24
CAMK2G	CCC-CCA-GGC-CCC-GCC-AGT-CCC-CCC-CCC-CGC-CCG-GCC-CCC-GGC-CCG-CCC-CC	10	50
DAP	CCC-CCG-CCC-CCG-CCC-CCG-CCC-CCG-CCC-CC	5	29
DRP2	CCC-CCT-CTT-CCC-CTC-TCC-CCC-TCT-CCC-CCT-CTC-TCC-CTC-TTC-CCC-CTC-TCC-TTG-TCT-CCTTCT-CTC-CCC-C	X	70
DUX4L22	CCC-CCG-AAA-CGC-GCC-CCC-CTC-CCC-CCT-CCC-CCC-TCT-CCC-CC	10	41
GH2	CCC-CCA-CCC-CCA-CCC-CCA-TCC-CCA-CGC-CCC-GCC-CCC-GCC-CCC	17	42
HIC2	CCC-CCG-GGA-CAG-GGA-CCC-TGG-CCC-CCC-CCG-ACA-GGC-TGA-CGC-CCA-CCC-CCT-CAA-ACT-CTG-GTG-GAC-TTA-CCC-CC	22	74
HOXC10	CCC-CCA-CCC-CCA-CCC-CCA-CCC-CCC	12	24
HOXD10	CCC-CCC-CCC-CCT-CCC-CCG-CGG-CCC-CC	2	26
JAZF1	CCC-CCC-CCG-CCC-CCG-CCC-CCG-CCC-TCC-CCC-C	7	31
LA16c-OS12.2	CCC-CCC-GTG-TCG-CTG-TTC-CCC-CCG-TGT-CGC-TGT-TCC-CCC-CGT-GTC-GCT-GTT-CCC-CCC	16	23
MSMO1	CCC-CCG-CCC-CCG-CCC-CCG-CCC-CC	4	45
NFATC1	CCC-CCG-TTT-CCC-CCG-CCA-GCC-CCA-GCG-CCC-CCC-TGC-CCG-GCC-CCC	18	45
PIM1	CCC-CCG-ACG-CGC-CCC-CCA-ACA-CAC-AAA-CCC-CCA-GAA-TCC-GCC-CCC	6	36
PLCB2	CCC-CCG-CCT-CTT-CTG-GAG-GCC-CCC-GCC-CCC-ACC-CCC	15	25
QSOX1	CCC-CCG-CCC-CCG-AGC-CCC-CGC-CCC-C	1	116
RAE1	CCC-CCC-GCC-CCC-CCC-GCC-CCC-CCG-CGC-CGC-CCC-CCC-CCG-CCC-CCC-GCC-CCC-GTC-CCC-CCG-CCC-CCC-CCG-CCC-CCC-CCG-CCC-CCC-GTC-CCC-CCG-CCC-CCC-CGC-CCC-CCC-GTC-CCC-CC	20	28
RUNX1-1	CCC-CCC-CCG-CAC-CCC-TTC-CCC-CGG-CCC-CCC-C	21	31
RUNX1-2	CCC-CCC-TCC-CCC-TGC-CTC-TCC-CTC-CCC-CCT-TTC-CCC	21	36
RUNX1-3	CCC-CCC-TTT-CCC-CTG-CCC-CCC-CTG-CCT-CCC-CC	21	32
SHANK1b	CCC-CCC-TCC-CCC-CAC-CCC-CCA-CCC-CCC-C	19	80
SHANK3	CCC-CCG-CCT-CCG-GCG-CAG-CCC-CCT-CGC-CAC-CCC-CGC-TTC-CCT-CCC-GTC-TCA-GGC-CCC-CTC-CCC-CCG-CCG-CCC-CCG-CCC-CC	22	79
SHANK3b	CCC-CCC-GCA-CCG-AGG-CCT-AGG-ACT-CCC-CCC-CCC-AAC-CCC-GTC-ACA-GCC-CCC-CAG-ACC-CCC-GCC-CCG-TGG-CTC-GGC-CCC-C	22	36
SNORD112	CCC-CCC-CCC-GCC-CCC-CAC-CCC-CCC-ACC-CCC-CCC-CCC	14	57
SOX1	CCC-CCT-GCA-GGC-CCC-CCT-GCG-CCT-CCC-CCC-CCC-CGC-CAC-TGG-CGC-CTG-GCT-TCC-CCC	13	33
STX17	CCC-CCG-CCC-CCG-CCC-CCG-CCC-CGC-AGG-GCC-CCC	9	57
TRABD	CCC-CCG-CCC-CCC-CCC-CCC-CCC-CC	22	23
WNT7A	CCC-CCG-CCC-CTC-CCT-CCT-TTC-CCC-CGT-CCC-TCC-CCC-GCC-CCC-TCC-CCC	3	48
ZBTB7B	CCC-CCC-ATC-CCT-CCC-CTC-CCT-CCC-CCC-GCC-CCT-GCC-ACC-CCC-CAA-ACT-CCC-CCC-CCC-C	1	50
ZFP41	CCC-CCA-GCC-CCC-GCC-GAC-CCC-CAG-CTC-CCG-CCT-CCG-CCG-ACC-CCC-AGC-CCC-C	8	52
ZNF480	CCC-CCG-CCC-CCG-CCC-CCG-CCC-CC	19	23

The thermal stability of the genomic oligonucleotide secondary structures were assessed as described previously using UV spectroscopy (Figure S9).

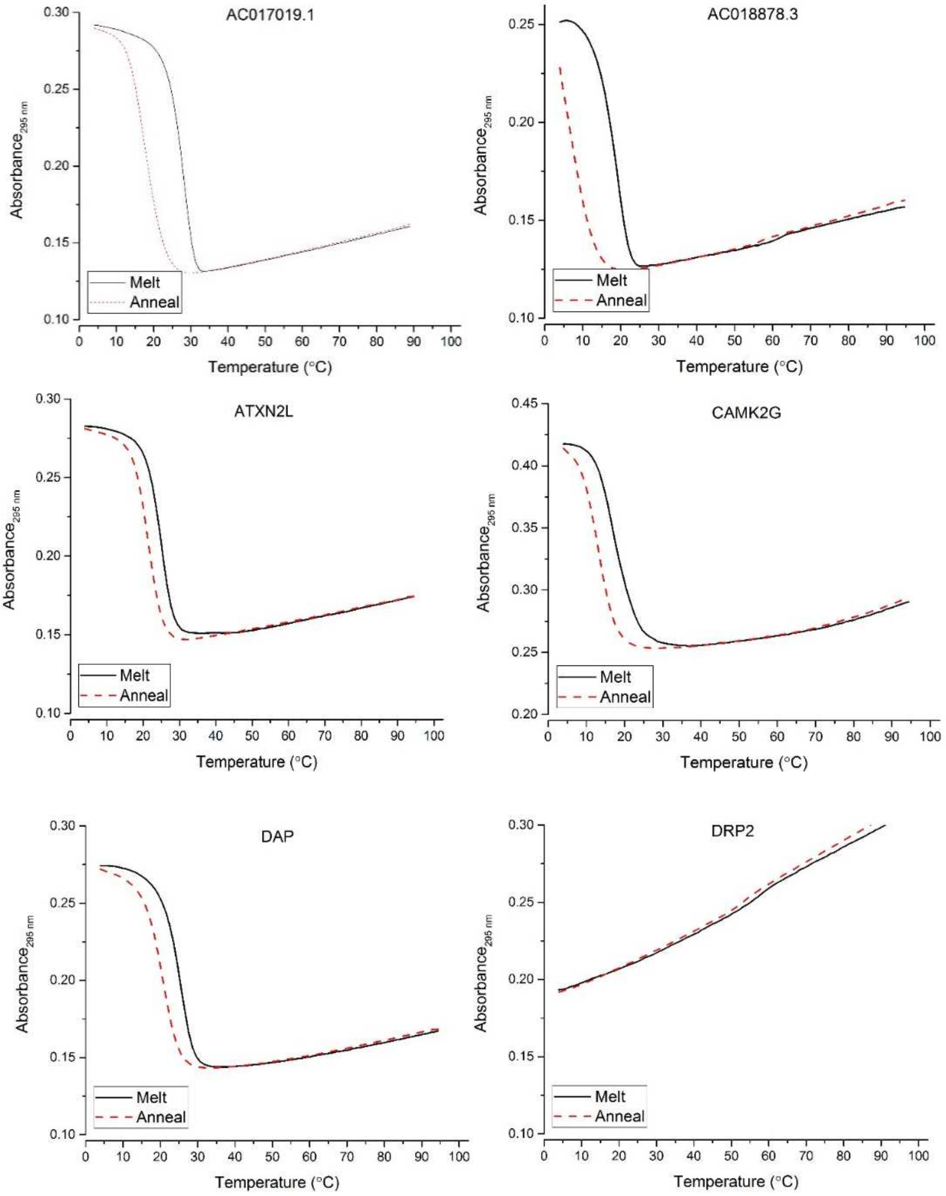

Figure S9A. The UV melt and annealing curves for the 33 genomic potential i-motif oligonucleotides. ODNs were diluted to 2.5 µM in 10 mM sodium cacodylate with 100 mM sodium chloride at pH 7.0.

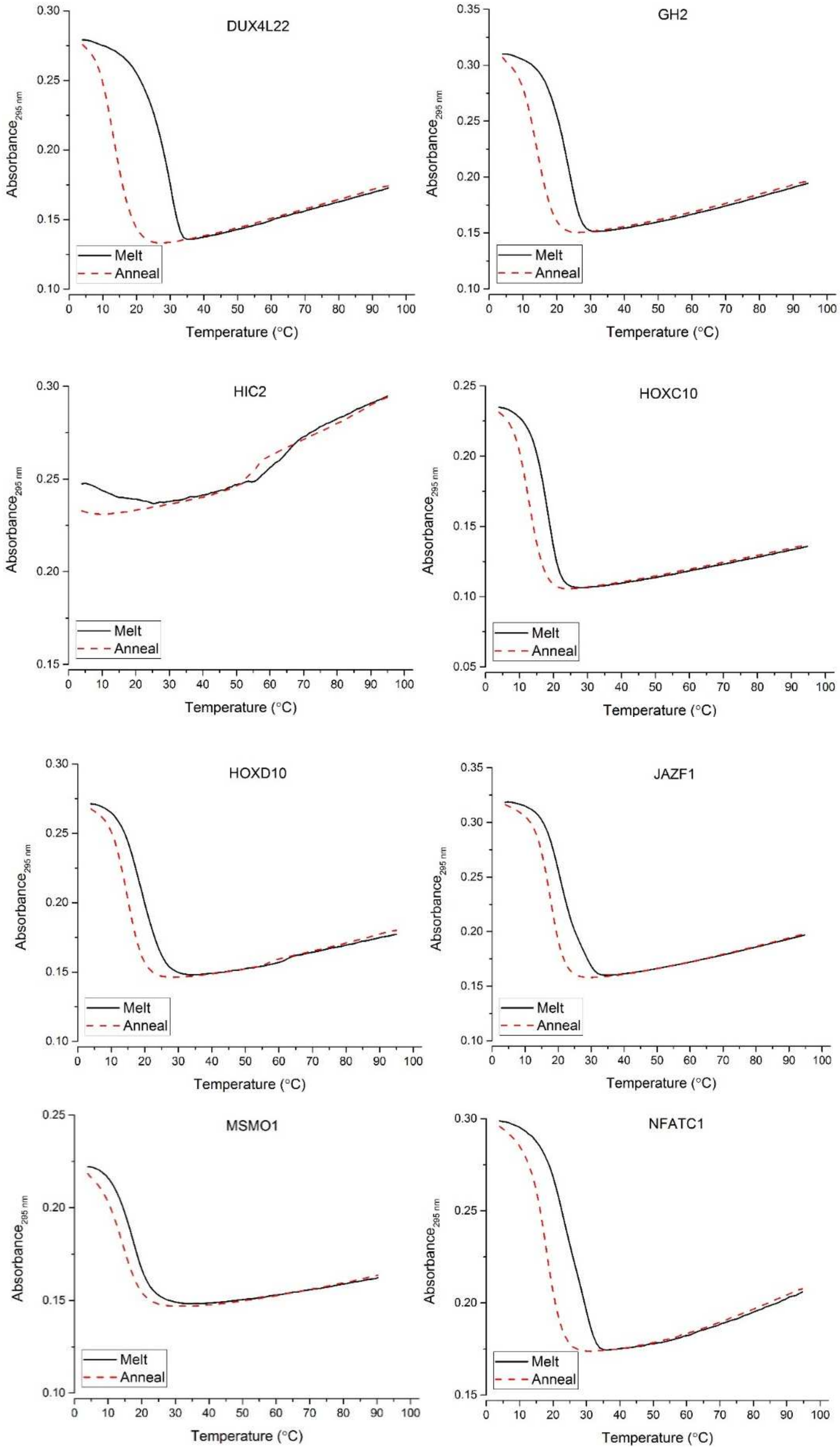

Figure S9B. The UV melt and annealing curves for the 33 genomic potential i-motif oligonucleotides. ODNs were diluted to 2.5 µM in 10 mM sodium cacodylate with 100 mM sodium chloride at pH 7.0.

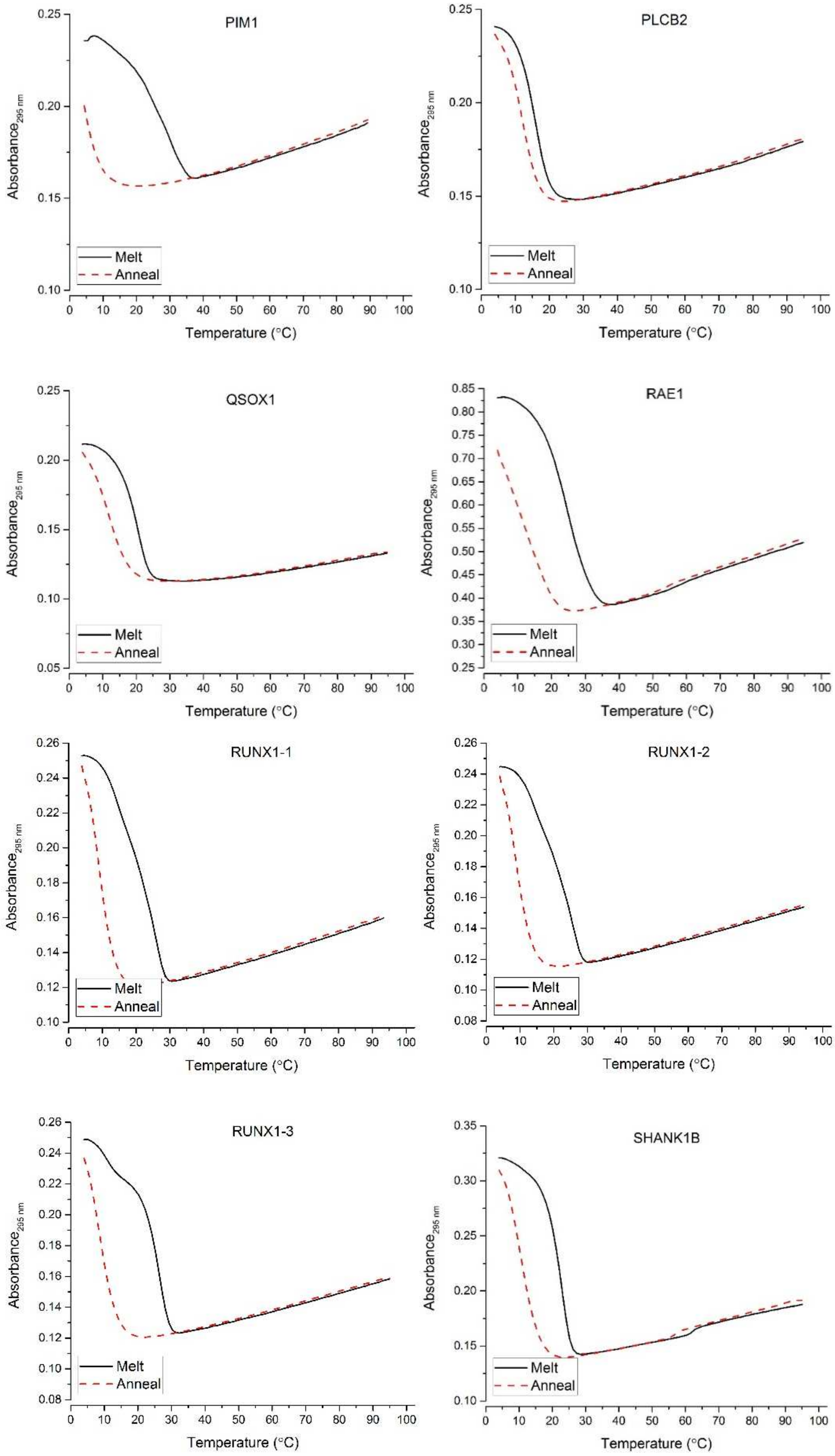

Figure S9C. The UV melt and annealing curves for the 33 genomic potential i-motif oligonucleotides. ODNs were diluted to 2.5 µM in 10 mM sodium cacodylate with 100 mM sodium chloride at pH 7.0.

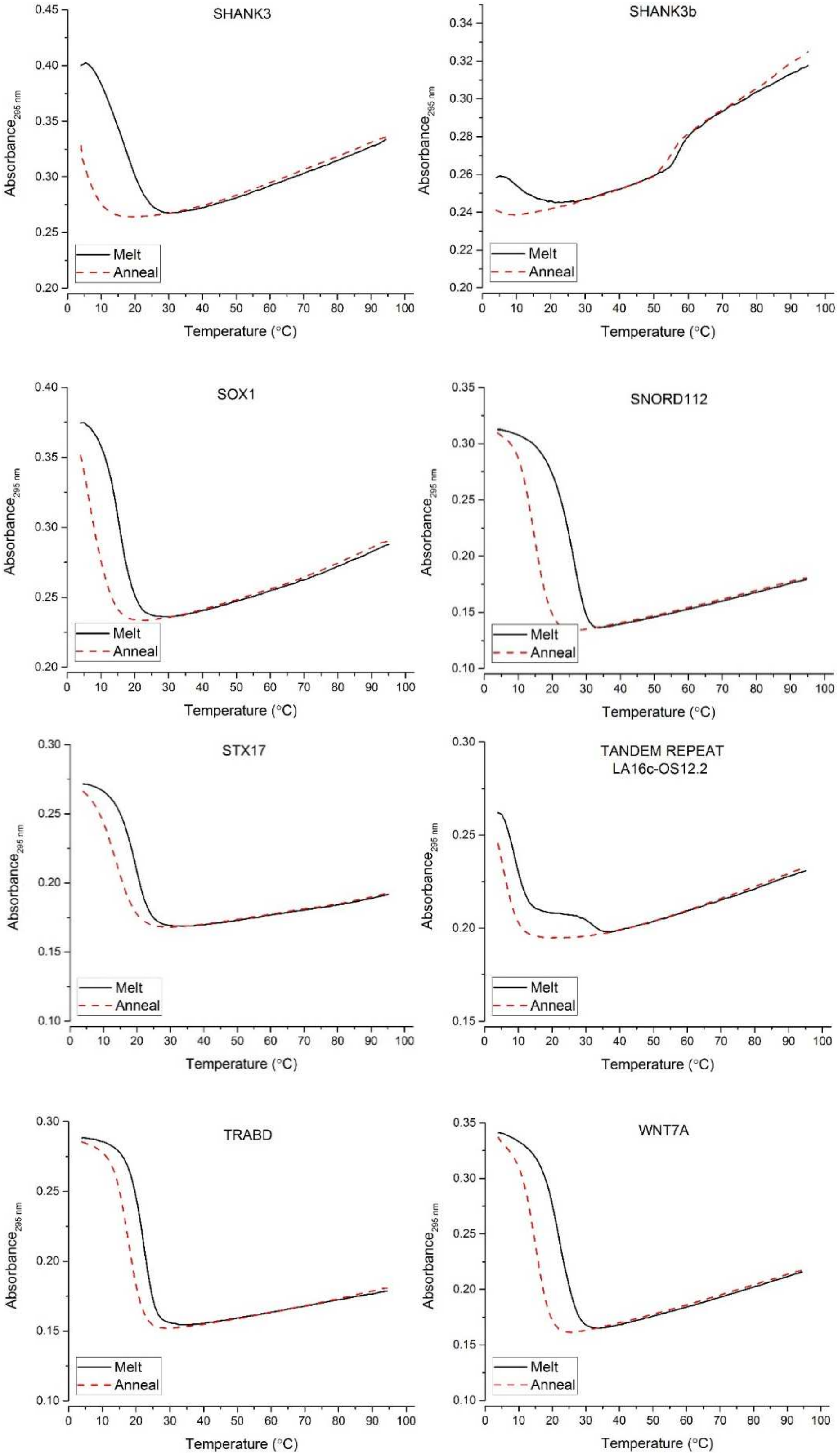

Figure S9D. The UV melt and annealing curves for the 33 genomic potential i-motif oligonucleotides. ODNs were diluted to 2.5 µM in 10 mM sodium cacodylate with 100 mM sodium chloride at pH 7.0.

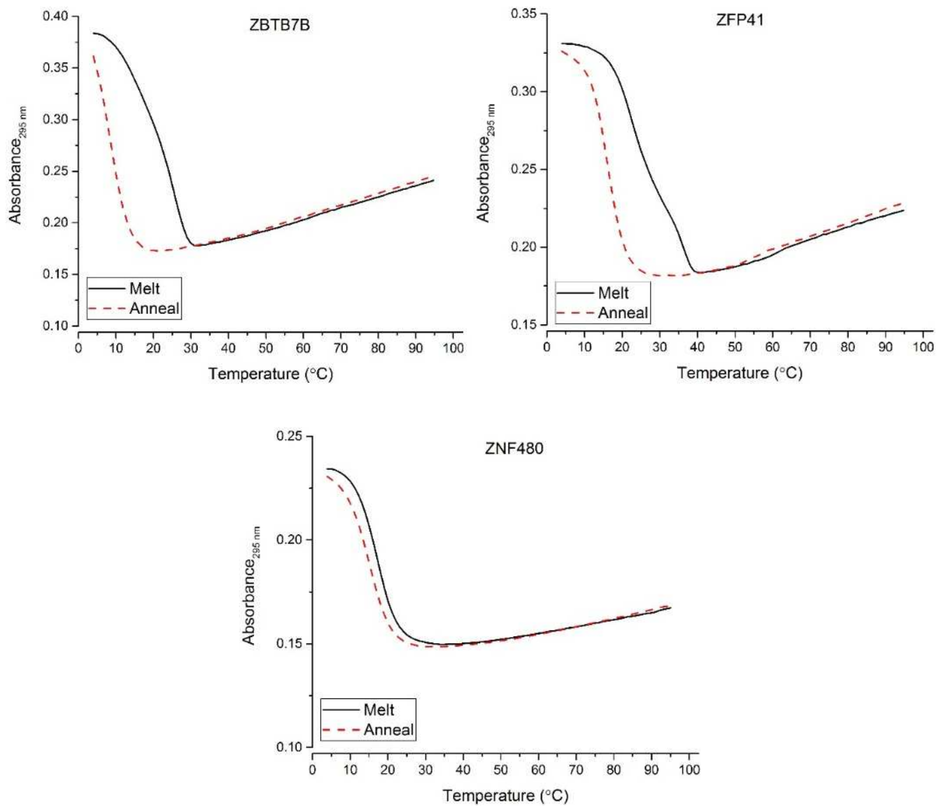

Figure S9E. The UV melt and annealing curves for the 33 genomic potential i-motif oligonucleotides. ODNs were diluted to 2.5 µM in 10 mM sodium cacodylate with 100 mM sodium chloride at pH 7.0.

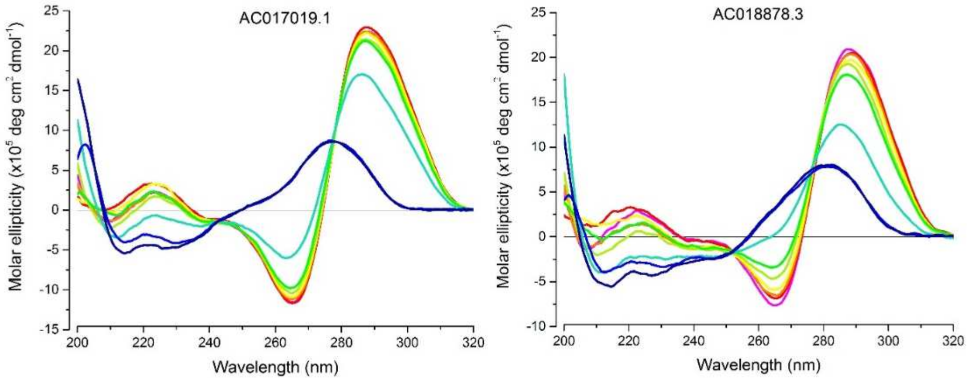

Figure S10A. The circular dichroism of each of the genomic potential i-motif ODNs (10 µM) measured in 10 mM sodium cacodylate with 100 mM sodium chloride at pH 4.0 to 8.0 in 0.5 pH unit increments. All signals were buffer subtracted. ■pH 4.0; ■pH 4.5; ■pH 5.0; ■pH 5.5; ■pH 6.0; ■pH 6.5; ■pH 7.0; ■pH 7.5; ■pH 8.0.

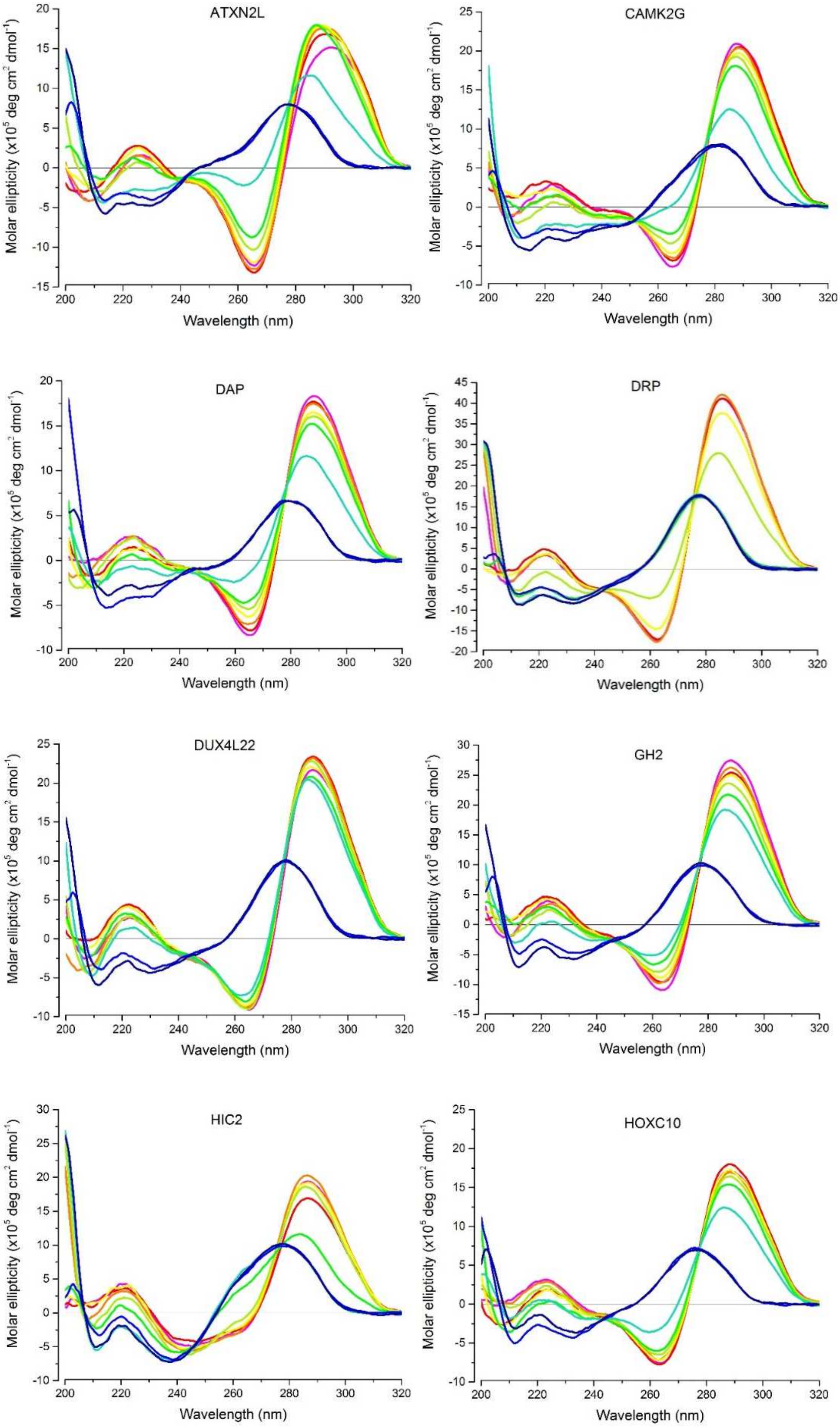

Figure S10B. The circular dichroism of each of the genomic i-motif ODNs (10 μM) measured in 10 mM sodium cacodylate with 100 mM sodium chloride at pH 4.0 to 8.0 in 0.5 pH unit increments. All signals were buffer subtracted. ■pH 4.0; ■pH 4.5; ■pH 5.0; ■pH 5.5; ■pH 6.0; ■pH 6.5; ■pH 7.0; ■pH 7.5; ■pH 8.0.

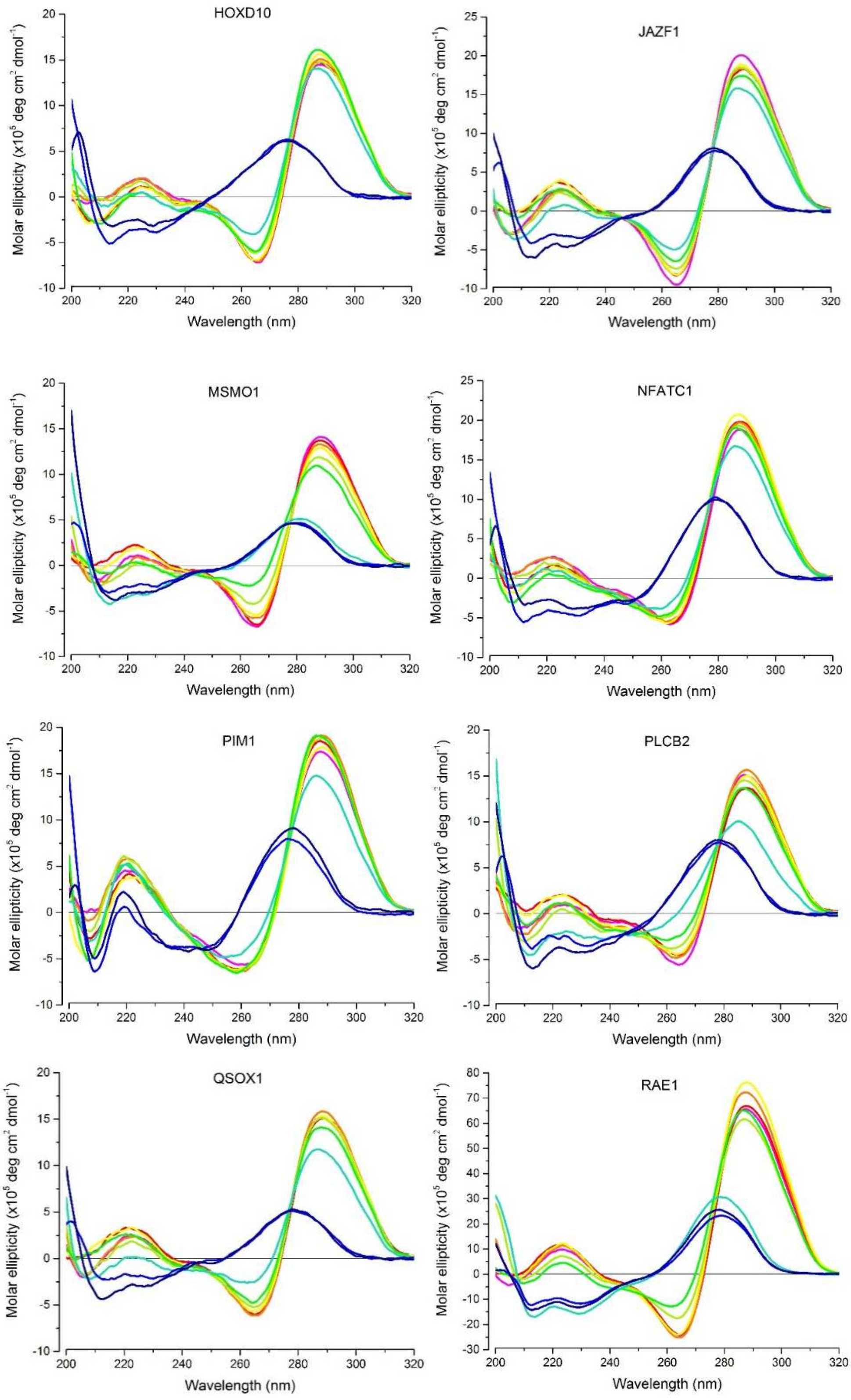

Figure S10C. The circular dichroism of each of the genomic i-motif ODNs (10 µM) measured in 10 mM sodium cacodylate with 100 mM sodium chloride at pH 4.0 to 8.0 in 0.5 pH unit increments. All signals were buffer subtracted. ■pH 4.0; ■pH 4.5; ■pH 5.0; ■pH 5.5; ■pH 6.0; ■pH 6.5; ■pH 7.0; ■pH 7.5; ■pH 8.0.

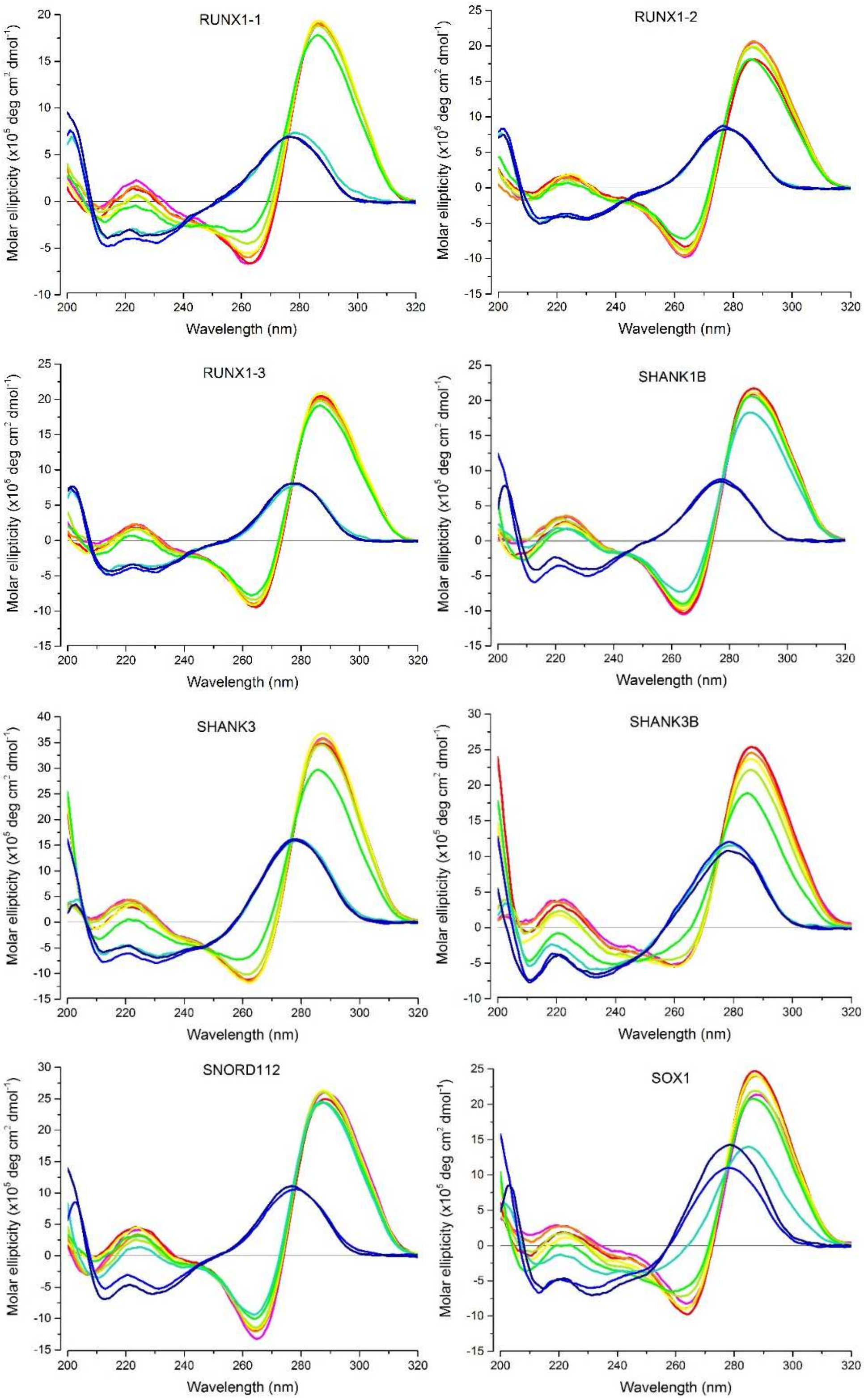

Figure S10D. The circular dichroism of each of the genomic i-motif ODNs (10 µM) measured in 10 mM sodium cacodylate with 100 mM sodium chloride at pH 4.0 to 8.0 in 0.5 pH unit increments. All signals were buffer subtracted. ■pH 4.0; ■pH 4.5; ■pH 5.0; ■pH 5.5; ■pH 6.0; ■pH 6.5; ■pH 7.0; ■pH 7.5; ■pH 8.0.

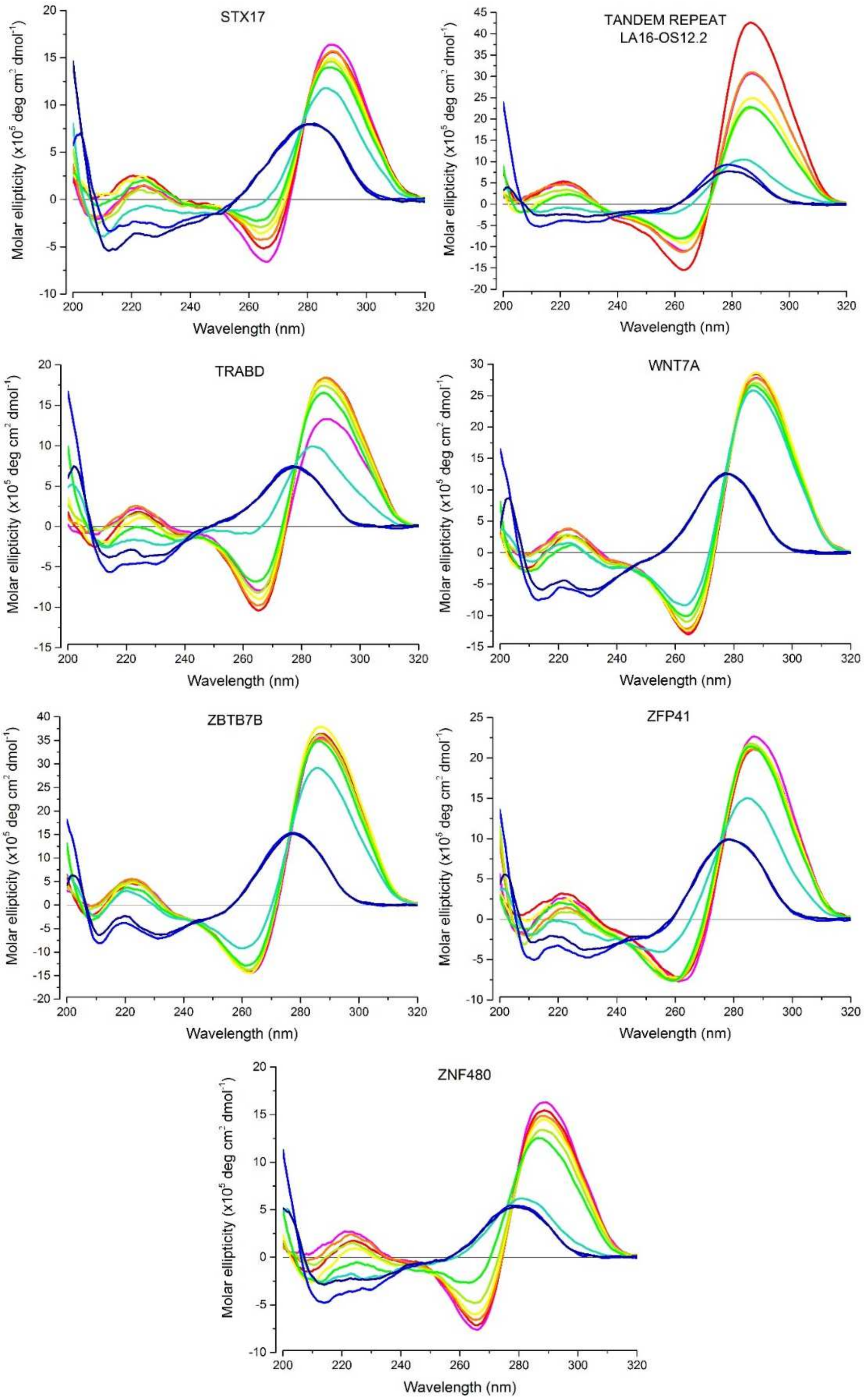

Figure S10E. The circular dichroism of each of the genomic i-motif ODNs (10 μM) measured in 10 mM sodium cacodylate with 100 mM sodium chloride at pH 4.0 to 8.0 in 0.5 pH unit increments. All signals were buffer subtracted. ■pH 4.0; ■pH 4.5; ■pH 5.0; ■pH 5.5; ■pH 6.0; ■pH 6.5; ■pH 7.0; ■pH 7.5; ■pH 8.0.

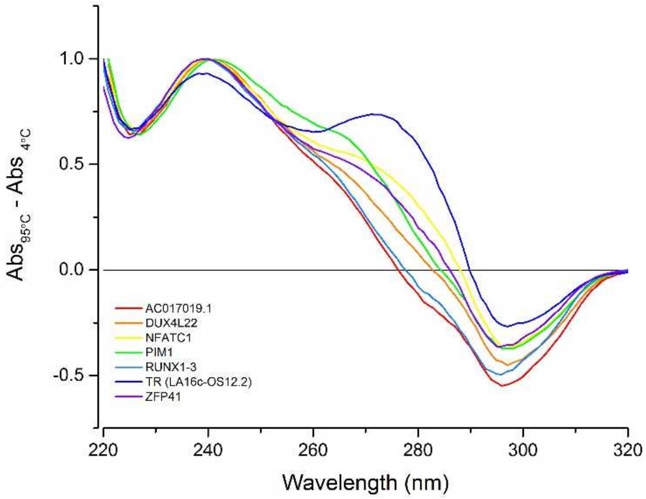

Figure S11. The thermal difference spectra calculated between 95 and 4°C for each of the ODNs (2.5 µM) with a $T_m \geq C_5T_3$ (26.2ºC) at pH 7.0. Samples were diluted in 10 mM sodium cacodylate with 100 mM sodium chloride.

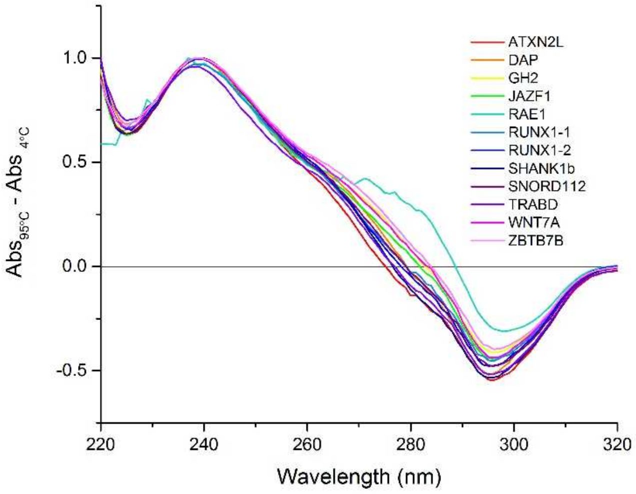

Figure S12. The thermal difference spectra calculated between 95 and 4°C for each of the ODNs (2.5 µM) with a $T_m \geq 20$°C at pH 7.0. Samples were diluted in 10 mM sodium cacodylate with 100 mM sodium chloride.
